# Functional Neuroanatomy of the Rat Nucleus Incertus–Medial Septum Tract: Implications for the Cell-Specific Control of the Septohippocampal Pathway

**DOI:** 10.3389/fncel.2022.836116

**Published:** 2022-02-25

**Authors:** Agata Szlaga, Patryk Sambak, Aleksandra Trenk, Anna Gugula, Caitlin E. Singleton, Gniewosz Drwiega, Tomasz Blasiak, Sherie Ma, Andrew L. Gundlach, Anna Blasiak

**Affiliations:** ^1^Department of Neurophysiology and Chronobiology, Institute of Zoology and Biomedical Research, Jagiellonian University, Krakow, Poland; ^2^The Florey Institute of Neuroscience and Mental Health, The University of Melbourne, Parkville, VIC, Australia; ^3^Florey Department of Neuroscience and Mental Health, The University of Melbourne, Parkville, VIC, Australia; ^4^Department of Anatomy and Physiology, The University of Melbourne, Parkville, VIC, Australia

**Keywords:** nucleus incertus, medial septum, relaxin-3, cholecystokinin, calcium-binding protein, electrophysiology

## Abstract

The medial septum (MS) is critically involved in theta rhythmogenesis and control of the hippocampal network, with which it is reciprocally connected. MS activity is influenced by brainstem structures, including the stress-sensitive, nucleus incertus (NI), the main source of the neuropeptide relaxin-3 (RLN3). In the current study, we conducted a comprehensive neurochemical and electrophysiological characterization of NI neurons innervating the MS in the rat, by employing classical and viral-based neural tract-tracing and electrophysiological approaches, and multiplex fluorescent *in situ* hybridization. We confirmed earlier reports that the MS is innervated by RLN3 NI neurons and documented putative glutamatergic (vGlut2 mRNA-expressing) neurons as a relevant NI neuronal population within the NI–MS tract. Moreover, we observed that NI neurons innervating MS can display a dual phenotype for GABAergic and glutamatergic neurotransmission, and that 40% of MS-projecting NI neurons express the corticotropin-releasing hormone-1 receptor. We demonstrated that an identified cholecystokinin (CCK)-positive NI neuronal population is part of the NI–MS tract, and that RLN3 and CCK NI neurons belong to a neuronal pool expressing the calcium-binding proteins, calbindin and calretinin. Finally, our electrophysiological studies revealed that MS is innervated by A-type potassium current-expressing, type I NI neurons, and that type I and II NI neurons differ markedly in their neurophysiological properties. Together these findings indicate that the MS is controlled by a discrete NI neuronal network with specific electrophysiological and neurochemical features; and these data are of particular importance for understanding neuronal mechanisms underlying the control of the septohippocampal system and related behaviors.

## Introduction

The septohippocampal pathway, most extensively studied in rodents, consists of projections between the medial septum (MS) and hippocampus, and is critically involved in theta rhythmogenesis and the control of hippocampal network excitability to fine-tune it to different behavioral states (Müller and Remy, 2018). The importance of the MS in theta rhythm generation is well established. For example, MS lesion or inactivation eliminates theta rhythm from all cortical targets (Buzsáki, 2002). MS neurons form a tightly interconnected network, in which three major cell types can be distinguished: (i) slow firing, theta-independent cholinergic neurons; (ii) cluster or slow-firing glutamatergic neurons, with activity correlated with running speed; and (iii) theta-modulated, fast and burst-firing GABAergic neurons (Sotty et al., 2003; Leão et al., 2015; Müller and Remy, 2018). Septal GABAergic and glutamatergic neurons innervate predominantly GABAergic neurons in the hippocampus, while the main targets of septal cholinergic projections are hippocampal pyramidal neurons (Sun et al., 2014).

MS neurons are under modulatory influences from several brain areas, including ascending cholinergic projections from the dorsolateral tegmental nucleus, serotonergic projections from the raphe nuclei, adrenergic inputs from the locus coeruleus (Takeuchi et al., 2021) and a dense projection from the brainstem, *nucleus incertus* (NI), an enigmatic structure involved in the control of contextual memories, locomotor speed, arousal, and stress responses (Ma et al., 2013, 2017a; Szőnyi et al., 2019; Lu et al., 2020).

NI is a bilateral group of neurons in the periventricular gray below the fourth ventricle (4V), with a midline part with densely packed neurons (NI *pars compacta*, NIc) and lateral parts with dispersed neurons (NI *pars dissipata*, NId), identified and studied in several mammalian species, including rat, mouse, and macaque (see Ma et al., 2017b for review). NI projects widely throughout the rat brain, but its most dense efferent projections terminate in the MS, closely related to hippocampal theta rhythmogenesis (Goto et al., 2001; Olucha-Bordonau et al., 2003). Notably, among structures innervated by the NI are the interpeduncular nucleus, median raphe, and supramammillary nucleus, regions also associated with orchestrating hippocampal neuronal activity in rats and mice (Olucha-Bordonau et al., 2003; Lu et al., 2020; Nasirova et al., 2020). The functional importance of NI connections has been demonstrated in several studies with a strong influence of NI manipulations on theta rhythm and associated locomotor activity and arousal-related processes. It was shown that electrical stimulation of rat NI induced, and NI lesion attenuated, brainstem-induced hippocampal theta rhythm (Nuñez et al., 2006) and chemogenetic activation of rat NI neurons enhanced locomotor activity and promoted arousal (Ma et al., 2017a). Recently, research in mice employing optogenetic stimulation of NI GABAergic neurons revealed that the NI controls hippocampal network state (Szőnyi et al., 2019), and selective activation and inhibition of NI neuromedin-B (NMB)-containing GABAergic neurons produced increased and decreased locomotor speed, arousal levels, and hippocampal theta power, respectively (Lu et al., 2020). These findings further indicate a central role for the NI in control of theta rhythm and related behaviors via actions within the septohippocampal network. Despite this, the anatomical, neurochemical, and electrophysiological properties of neuronal populations within the NI, including those that comprise the NI-MS tract, remain imprecisely defined.

In the rat, NI projection neurons express the GABA-synthesizing enzyme, glutamate decarboxylase (GAD) (Olucha-Bordonau et al., 2003), and several neuropeptides, including, relaxin-3 (RLN3), which is strongly colocalized with GAD (Ma et al., 2007). NI is the major source of RLN3 in the rat brain (Ma et al., 2017b), but within this structure other neuropeptides and neurotransmitters, such as cholecystokinin (CCK), NMB, substance P, galanin, dynorphin, and glutamate (Chronwall et al., 1985; Sutin and Jacobowitz, 1988; Olucha-Bordonau et al., 2003; Ma et al., 2007), have been identified. Furthermore, the presence of the calcium-binding proteins; calbindin and calretinin (but not parvalbumin) was confirmed in the NI (Paxinos, 1999; Cervera-Ferri et al., 2012).

NI neurons are diverse not only in their neurochemistry, but also in their neurophysiology, and under *ex vivo* conditions, we distinguished neurons with different electrophysiological characteristics within this structure—type I neurons comprising RLN3-expressing cells, characterized by the presence of an A-type potassium current (I_A_), and type II neurons expressing a low voltage-activated, nickel-sensitive putative T-type calcium current (Blasiak et al., 2015). However, the detailed electrophysiological characteristics of NI neurons, including those neurons innervating the MS, remain unknown.

It was shown in rats that RLN3 and glutamate-synthetizing NI neurons innervate the MS (Cervera-Ferri et al., 2012; Olucha-Bordonau et al., 2012), and that activation of the RLN3 cognate receptor, relaxin-family peptide receptor 3 (RXFP3) (Liu et al., 2003) in the MS enhanced hippocampal theta rhythm and spatial memory (Ma et al., 2009). Within the MS, RXFP3 is expressed by GABAergic and glutamatergic, but not cholinergic neurons (Albert-Gascó et al., 2018; Haidar et al., 2019), and their presence is necessary for proper learning and long-term memory formation (Albert-Gascó et al., 2018; Haidar et al., 2019). Collectively, these data indicate the importance of the NI in the control of the septohippocampal axis and reveal the possible neuroanatomical basis of the influence of the NI and the RLN3/RXFP3 system on hippocampal theta rhythm at the level of the MS.

Despite the role of the NI in the control of hippocampal theta rhythm, arousal, and locomotor activity, a better knowledge of the neurotransmitter and electrophysiologically-defined cell types within this region is still required. Therefore, this study aimed to establish the neurochemical and electrophysiological profile of NI neurons in the rat brain, with a special focus on MS-projecting cells. With the use of optogenetic tagging of NI-MS neurons, and a combination of retrogradely-transported AAV vectors and multiplex fluorescent *in situ* hybridization (RNAscope), *ex vivo* patch-clamp electrophysiology, neural tract-tracing and immunohistochemistry, we documented the characteristics of NI neurons and the NI–MS axis, and have generated an important data resource for use in designing future studies.

## Materials and Methods

### Ethical Approval

Procedures were conducted in accordance with the directive 2010/63/EU of the European Parliament and of the Council of September 22, 2010 on the protection of animals used for scientific purposes, and ethical guidelines of the National Health and Medical Research Council of Australia. Procedures involving neural tract-tracing and experiments with viral vector injections were also conducted in accordance with the Polish Act on the Protection of Animals Used for Scientific or Educational Purposes of January 15, 2015 and approved by the 2nd Local Institutional Animal Care and Use Committee (Krakow, Poland), approval number 24/2021. Immunohistochemical experiments were conducted with the approval of The Florey Institute of Neuroscience and Mental Health Animal Ethics Committee. All efforts were made to minimize suffering and to reduce the number of animals used.

### Animals

Experiments were conducted using male, Sprague-Dawley rats—those used in immunohistochemical experiments were obtained from the Animal Resources Centre (Canning Vale, WA, Australia); while the remainder were obtained from the Institute of Zoology and Biomedical Research (Jagiellonian University, Krakow, Poland). Rats were kept in plastic cages lined with wooden bedding, under constant temperature conditions (21 ± 2°C), and were maintained on a 12-12 light-dark cycle with *ad libitum* access to fresh water and standard laboratory rodent chow.

### Stereotaxic Injection of Viral Vectors

Seven-week-old rats (*n* = 11) for electrophysiological studies and 12 week-old rats (*n* = 3) for anatomical studies were anesthetized with an intraperitoneal injection of a mixture of ketamine (100 mg/kg body weight, Ketamina; Biowet, Pulawy, Poland) and xylazine (10 mg/kg body weight, Sedazin; Biowet), with supplementary injections of ketamine (30 mg/kg), as required. Prior to surgery, an anti-inflammatory drug, tolfedine, was injected subcutaneously (0.1 ml/100 g body weight, Vétoquinol Biowet, Gorzow Wielkopolski, Poland). Throughout the procedure, body temperature was maintained at 37 ± 0.5°C (temperature controller TCP-02, WMT, Krakow, Poland), and the eyes were protected from drying by the application of eye drops (Starazolin HydroBalance, Polpharma, Starogard Gdanski, Poland). Rats were placed into a stereotactic frame (ASI Instruments Inc., Warren, MI, United States), a midline scalp incision was made, and a craniotomy was drilled over the MS (∼1 mm diameter). A glass microcapillary tube (VITREX, Herlev, Denmark) was prepared on a vertical puller (PE-21, Narishige Scientific Instrument Laboratory, Tokyo, Japan) to obtain an ultra-thin tip that was then broken to a final diameter of 40–50 μm. The injection needle was tightly connected to a Hamilton syringe (1 μl, Hamilton, Bonaduz, Switzerland) with tygon tubing (IDEX Health and Science GmbH, Wertheim, Germany), and the system was filled with paraffin oil (Sigma-Aldrich, Poznan, Poland). Just before the injection, the tip of the injection needle was filled with a canine adenovirus engineered to express Cre-recombinase (CAV2-CMV-Cre, 2 injections, 300 nl per injection, 1.5 × 10^9^ gene copies, Plateforme de Vectorologie de Montpellier, Montpellier, France) or retrograde pAAV-hSyn-mCherry viral vectors, produced with the rAAV2-retro helper plasmid (2 injections, 300 nl per injection, 4.2 × 10^9^ gene copies, Addgene, Teddington, United Kingdom). The coordinates relative to bregma used for MS injections were as follows: AP: + 0.72 mm, ML: ± 0.00 mm, DV: –6.50 and –7.40 mm. Viral vectors were delivered with the speed of 1 nl/s.

Subsequently, in rats that received MS injections of CAV2-CMV-Cre, the bregma point was set below the level of the lambda point (usually 1.8 mm) to obtain a rostral inclination of the skull of 15°, allowing the needle to bypass the confluence of the superior sagittal sinus and transverse sinuses, when descending into the brain. A small craniotomy (∼1 mm diameter) was drilled above the NI. A second Hamilton syringe and injection needle prepared in a similar manner as described, was used to inject a Cre-dependent adenovirus carrying the gene for channelrhodopsin-2 (ChR2) and the mCherry fluorescent protein [AAV2-EF1a-DIO-ChR2(H134R)-mCherry; 2 injections, 200 nl per injection, 2 × 10^9^ gene copies, Addgene]. The coordinates relative to lambda used for NI injections were as follows: AP: –2.2 mm, ML: ± 0.15 mm, DV: –6.3 mm.

Finally, the surgical incision was stitched and covered with spray containing aluminum ions (Alu-spray, MEDiVET, Krakow, Poland) to promote healing. After surgery, rats were allowed to recover for 2 weeks, and their condition was monitored.

### Multiplex Fluorescent *in situ* Hybridization (RNAscope)

In studies to determine the neurochemical nature of NI neurons innervating the MS, *in situ* hybridization using an RNAscope HiPlex Assay [Advanced Cell Diagnostics (ACD), Hayward, CA, United States] with RNAscope HiPlex Alternate Display Module (ACD; for AF488, Atto550 and Atto647 detection) was conducted on brain sections from 3 male Sprague-Dawley rats, 4 weeks after the injection of the retrograde viral vector encoding mCherry protein, into the MS. All procedures were performed following the manufacturer’s instructions, with preparation and pretreatment for fresh frozen samples. Briefly, rats were deeply anesthetized with isoflurane (Aerrane; Baxter, Warsaw, Poland) and decapitated. Their brains were immediately collected, frozen on dry ice and stored at –80°C. For each brain, three 16 μm sections containing NI were cut at –20°C, using a cryostat (Cryocut CM 1800, Leica Microsystems, Wetzlar, Germany), and mounted onto Superfrost-Plus slides (Thermo Fisher Scientific, Braunschweig, Germany). The slides were stored at –80°C until a 1 h fixation in a freshly-prepared solution of 4% formaldehyde in PBS (pH 7.4, initially 4°C) at room temperature (RT), followed by washing in PBS and dehydration in ethanol solutions of increasing concentration (50, 70, and 100%). Dehydrated sections were stored at –20°C overnight and the next day were air-dried, outlined with Immedge Hydrophobic Barrier Pen (Vector Laboratories, Burlingame, California, United States) and incubated with Protease IV pretreatment solution (ACD) for 30 min at RT. After washing in PBS, the sections were hybridized for 2 h at 40°C with a solution of HiPlex probes for Round 1: RLN3 (Rn-Rln3-T1, cat. no. 1037211-T1, ACD), mCherry (mCherry-T2, cat. no. 431201-T2, ACD), vGAT1 (Rn-Slc32a1-T3, cat. no. 424541-T3, ACD), CRHR1 (Rn-Crhr1-T4, cat. no. 318911-T4, ACD), and vGlut2 (Rn-Slc17a6-T5, cat. no. 31701-T5, ACD) in HiPlex Probe Diluent (ACD). Next, hybridization with signal amplifying reagents (HiPlex Amp 1, Amp 2, and Amp 3, ACD; 40°C, 30 min each) was performed and at the end of Round 1, the sections were hybridized with HiPlex Fluoro T1–T3 (ACD). Sections were washed in 1×Wash Buffer (ACD) between every hybridization step. Finally, the tissue was counterstained with DAPI, coverslipped with ProLong Gold antifade reagent (Invitrogen, Thermo Fisher Scientific, Life Technologies Corporation, Eugene, OR, United States) and imaged for Round 1 (probes T1–T3 and DAPI) using an Axio Imager M2 fluorescent microscope (Zeiss) with an automatic z-stage and Axiocam 503 mono camera (Zeiss), equipped with 20×/0.5 EC Plan Neo-Fluar objective for acquisition of panoramic z-stack images of the whole NI (scaling: 0.227 μm in x and y, and 1.250 μm in z axis) and 40×/1.3 Oil EC Plan Neofluar objective for obtaining single representative z-stack images of NIc and NId regions (scaling: 0.114 μm in x and y, and 0.280 μm in z). The next day, slides were soaked in 4×SSC buffer (prepared using UltraPure 20×SSC Buffer, cat. no. 15557-044, Invitrogen, Life Technologies Limited, Paisley, United Kingdom) at RT for 2 h, to enable safe cover glass removal. To cleave Round 1 fluorophores, the sections were incubated with 10% TCEP cleaving buffer prepared with the Cleaving Stock Solution (ACD) and 4×SSC, twice for 15 min (RT), alternated with washing with fresh PBST (PBS mixed with 0.5% Tween-20^®^, Sigma-Aldrich). Thereafter, the sections were hybridized with HiPlex Fluoro T4-T6 (ACD) for 15 min at 40°C, re-coverslipped with ProLong Gold antifade reagent and imaged for Round 2 (probes T4–T5 and DAPI), as described for Round 1.

All acquired images were processed in Zen software (3.1 blue edition and 3.0 SR black edition, Zeiss) to improve the signal-to-noise ratio and convert them into maximum intensity projection images. Panoramic (20×) images underwent additional tile alignment using CorelDraw 2020 software (Ottawa, Canada). Finally, images of the same regions of interest for both rounds were matched, combined with HiPlex Image Registration software (ACD), using DAPI as a reference and saved as merged RGB, as well as single channel grayscale images. The latter (3 sections from rat 1 and 2, and 2 sections from rat 3) were then used for counting mCherry-positive cells located in NI, with an ImageJ Cell Counter plugin (Schneider et al., 2012). During this process, co-expression (juxtaposition within one cell) of mCherry with other examined mRNA molecules (for RLN3, vGAT1, vGlut2, and CRHR1) was determined. Cells were identified by the presence of the nucleus and/or an unambiguous cell-like distribution of fluorescent mRNA dots. A cell was classified as expressing a given mRNA when at least three clear dots of specific fluorescence were present within its boundary. Heatmaps and graphs representing the distribution and density of the various types of mCherry-positive cells in the NI were generated using a custom script in Python.

### Stereotaxic Injection of a Retrograde Tracer

Adult Sprague-Dawley rats (*n* = 6; 14 weeks old) were deeply anesthetized in an induction chamber filled with a 3–4% (v/v) mixture of isoflurane and air (Aerrane; Baxter). Anti-inflammatory and analgesic drugs were injected subcutaneously (Tolfedine; 0.4% in saline; 0.1 ml/100 g body weight; Vétoquinol Biowet, and intramuscularly (Torbugesic; 0.1% in saline; 0.01 ml/100 g body weight; Zoetis, Warsaw, Poland). Rats were placed in the stereotaxic apparatus (SAS-4100; ASI Instruments) equipped with a rat gas anesthesia mask (Stoelting, Wood Dale, IL United States) that allowed the rat to breathe air with 2–3% (v/v) concentration of isoflurane. The body temperature was held at 37 ± 0.5°C (temperature controller TCP-02; WMT) and the depth of anesthesia was monitored during the surgery, by checking the persistent disappearance of the corneal reflex and withdrawal response to paw pinch. A small skin incision was made, and based on stereotaxic coordinates (Paxinos and Watson, 2007), a craniotomy was completed. Injection pipettes were prepared on the vertical puller (PE-21, Narishige Scientific Instrument Laboratory) from glass capillaries (VITREX). The injection needle connected to a Hamilton syringe (1 μl; Hamilton) with tygon tubing (IDEX), was filled with paraffin oil (Sigma-Aldrich) and just before lowering into the MS, the tip of the microinjection capillary was backfilled with FluoroPink tracer (Tombow Pencil Co., Tokyo, Japan). The following coordinates were used for MS injections: AP: + 0.6 mm, ML: 0 mm, DV: –7.2 and –6.6 mm ventral from bregma. At each ventral point, 300 nl of the tracer were injected. Tracer was injected manually at a speed of 50 nl/min. To minimize reflux of injectate up the cannula, the needle was left in place for 10 min and then withdrawn. The scalp was sutured using surgical silk and the wound was covered with spray-dressing (Nanosilver, Bioton, Macierzysz, Poland) to promote healing. Rats were left to recover from the anesthesia in a heated chamber and their health condition was monitored daily for 1 week.

### Stereotaxic Injection of Colchicine

Colchicine leads to the accumulation of peptides and all other transported material in neuronal soma by disrupting axonal transport, and can be used to improve the quality of immunohistochemical staining in the soma of neurons (Kreutzberg, 1969). Therefore, a colchicine pre-treatment protocol was used in the neural tract-tracing and immunohistochemical studies to characterize NI neurons. A similar procedure as described above was conducted for rats (*n* = 6) injected with retrograde tracers, 1 week after the first surgery, and for a separate group of rats (*n* = 5). A unilateral, intracerebroventricular administration of colchicine solution (Sigma-Aldrich) was made at the following coordinates for the neural tract-tracing studies: AP: –0.7 mm, ML: 1.8 mm, DV: –4.0 mm from bregma (200 μg in 5 μl sterile saline); or at AP: –0.2 mm, ML: 1.5 mm, DV: –4 mm from bregma (80 μg in 5 μl) for the immunohistochemical studies.

### Histology and Immunostaining

All colchicine-treated rats were perfused with fixative 24 h after the icv administration. Rats were deeply anesthetized with pentobarbital (100 mg/kg, i.p.) and transcardially-perfused with 300 ml of ice-cold 0.1 M phosphate-buffered saline (PBS) followed by 400 ml of 4% formaldehyde in PBS. For the neural tract-tracing studies, the brains were extracted and kept overnight in 4% formaldehyde solution. The fixed brains were cut into 50 μm coronal slices on a vibrating microtome (VT1000S; Leica, Heidelberg, Germany). The retrograde-tracer injection efficiencies were histologically verified, and one brain was excluded from further procedures, due to leakage of tracer to the corpus callosum. From the correctly injected brains, every second slice containing the NI was used for the immunohistochemical procedure. Brains from rats without retrograde tracer injections, were dissected and submerged in 30% sucrose in PBS for 48 h at 4°C. Coronal sections (30 μm) through the rostrocaudal extent of the brainstem, from caudal DR (bregma –8.4 mm), through the NI, to the anterior *prepositus nucleus* (bregma –10.4 mm) were collected free-floating into PBS in three series.

Sections were incubated in blocking buffer (10% v/v NDS (Jackson ImmunoResearch, West Grove, PA, United States) in PBS with 0.6% Triton-X for the tract-tracing studies or 10% v/v NGS (Jackson ImmunoResearch) in PBS with 0.1% Triton-X for the immunohistochemical studies) for 1.5 h with agitation at RT. Sections were then incubated for 16–72 h in PBS containing multiple primary antibodies (Supplementary Table 1), 2% NDS, and 0.3% Triton-X for tract-tracing studies or 2% NGS, and 0.1% Triton-X for immunohistochemical studies. Sections were washed 3 × 10 min followed by incubation in dilutions of Alexa Fluor-conjugated secondary antibodies (1:400 for tract-tracing studies or 1:500 for immunohistochemical studies; Supplementary Table 2) in PBS overnight at 4°C or for 1 h at RT. For sequential immunofluorescence, sections were initially incubated with the first primary antibody, followed by washes and incubation in the appropriate secondary antibody, followed by 3 × 10 min in PBS and incubation with the second primary antibody and second secondary antibody. After final rinsing (3 × 5 min), sections were mounted on glass slides and coverslipped with Fluoroshield with DAPI (Sigma-Aldrich) for tract-tracing studies or Fluoromount-G (Southern Biotech, Birmingham, AL, United States) for immunohistochemical studies.

Slices from rats that received tracer injections were viewed and photographed using a confocal microscope (Axio Observer Z1; Zeiss, Gottingen, Germany). NI neurons labeled retrogradely and containing RLN-3 and CCK were counted manually using ZEN software (Zeiss) and represented as mean ± SD per section. The percentage of neurons displaying FluoroPink and the expression of RLN-3, CCK, or both was then calculated. Images of sections from other brains were acquired with a 780 Laser Scanning Confocal microscope (Zeiss, Oberkochen, Germany) using a 20× (1 μm slice) objective. The system was equipped with a stitching stage, and Zen software (Zeiss) was used to stitch tiled images of the NI and surrounding region. Quantification of clearly identified cell bodies and colocalization of immunofluorescence, in addition to NeuN nuclear staining in some experiments, were manually performed on sections through the NI from bregma –9 to –9.84 mm (6–8 sections per rat) using Fiji (Schindelin et al., 2012) and represented as mean ± SEM. Details of the primary and secondary antibodies used are presented in Supplementary Tables 1, 2, respectively.

### *Ex vivo* Electrophysiology

Whole-cell patch-clamp electrophysiological recordings were performed as described (Kania et al., 2020a). Seven-week-old, male Sprague-Dawley rats were deeply anesthetized with isoflurane (Baxter) and decapitated. Brains were collected in ice-cold, low-sodium, high-magnesium artificial cerebrospinal fluid (ACSF), containing (in mM): 185 sucrose, 25 NaHCO_3_, 3 KCl, 1.2 NaH_2_PO_4_, 2 CaCl_2_, 10 MgSO_4_, and 10 glucose, (pH 7.4; osmolality 290–300 mOsmol/kg) and cut into 250 μm coronal sections on a vibrating microtome (Leica). Sections containing the NI were transferred to an incubation chamber containing carbogenated, warm (32°C) ACSF, containing (in mM): 118 NaCl, 25 NaHCO_3_, 3 KCl, 1.2 NaH_2_PO_4_, 2 CaCl_2_, 1.3 MgSO_4_ and 10 glucose, (pH 7.4; osmolality 290–300 mOsmol/kg). After a recovery period (90–120 min, RT) slices were placed in a recording chamber, where the tissue was perfused (2 ml/min) with carbogenated, warm (32°C) ACSF of the same composition.

Recording micropipettes were fabricated from borosilicate glass capillaries (7–9 MΩ; Sutter Instruments, Novato, CA, United States) using a horizontal puller (Sutter Instruments) and filled with a solution containing (in mM): 145 potassium gluconate, 2 MgCl_2_, 4 Na_2_ATP, 0.4 Na_3_GTP, 5 EGTA, 10 HEPES (pH 7.3; osmolality 290–300 mOsmol/kg) and 0.05% biocytin (for subsequent immunofluorescent identification of recorded neurons). The calculated liquid junction potential was +15 mV and data were corrected for this value. All reagents for the PBS, ACSF, and intrapipette solutions were purchased from Sigma-Aldrich, apart from biocytin, which was purchased from Tocris Bioscience (cat.no. 3349, Tocris Bioscience, Bristol, United Kingdom).

NI neurons were located and approached using an Examiner D1 microscope (Zeiss) equipped with video-enhanced infrared differential interference contrast. Cell-attached and whole-cell configurations were obtained using a negative pressure delivered by mouth suction. SEC 05LX amplifier (NPI, Tamm, Germany), Micro 1401 mk II (CED, Cambridge Electronic Design, Cambridge, United Kingdom) converter and Signal and Spike 2 software (CED) were used for signal recording and data acquisition. Recorded signal was lowpass filtered at 3 kHz and digitized at 20 kHz.

#### Optogenetic Tagging and Tissue Processing

Brain slices from rats that received viral vector injections, were prepared for electrophysiological recordings 2 weeks after the surgery, as described above. mCherry expressed by NI neurons innervating the MS, was excited at 530 nm by LED illumination (Colibri, Zeiss). In order to further verify that recorded NI neurons were constituents of the NI-MS pathway, optogenetic tagging of recorded NI neurons was performed. The optical fiber (core diameter, 200 μm; NA, 0.5; Thorlabs, Newton, NJ, United States) was positioned above the slice, as close to the recorded neuron as possible. The optical fiber was connected to a blue light emitting LED (λ = 465 nm, PlexBright LED Module, Plexon Inc., Dallas, TX, United States). The power of the LED output was controlled by a precise current source (PlexBright LED Driver LD-1, Plexon Inc.) and never exceeded 8 mW, as measured with the optical power meter (PM-100D equipped with S121C photodiode, ThorLabs) at the tip of the optical fiber. The temporal parameters of the light pulses were digitally controlled by Spike 2 software running a custom written script (CED). At least 20 light pulses (5 ms, 0.1 Hz) were applied to each recorded NI neuron. All recordings were performed in ACSF containing the glutamate ionotropic receptor antagonists, CNQX (10 μM, cat. no. 1045, Tocris Bioscience) and DL-AP5 (50 μM, cat. no. 0105, Tocris Bioscience) and the GABA_A_ receptor antagonist, gabazine (5 μM, SR95531, Tocris Bioscience).

After the electrophysiological recordings, brain slices were processed as described in the histology and immunostaining section. Slices containing MS were incubated with a mouse anti-Cre recombinase antibody solution (1:1,000, cat. no. MAB3120, Sigma-Aldrich), followed by incubation with anti-mouse Alexa Fluor-488 antibodies (1:400, cat. no. 715-545-151, Jackson ImmunoResearch). Slices containing NI were incubated with a solution containing a rabbit anti-mCherry antibody (1:1,000, cat. no.167453, Abcam, Cambridge, United Kingdom) and subsequently with anti-rabbit Cy-3 antibodies (1:400, cat. no. 711-165-152, Jackson ImmunoResearch).

#### Electrophysiological Data Analysis

Electrophysiological properties of NI neurons were analyzed using custom Spike2 and Signal (CED) and MATLAB (The MathWorks, Natick, MA, United States) scripts. Current-clamp recordings to investigate spontaneous activity of NI neurons, were performed at zero holding current. NI neuron properties were calculated from voltage responses to current stimuli delivered from a membrane potential of –75 mV, sustained with continuous current injections. Action potential (AP) threshold and rheobase was assessed based on voltage responses to current ramp (0-1 nA, 1 s) application. Time to first AP were calculated from the first AP evoked by a depolarizing (+80 pA, 500 ms) current pulse. Excitability of recorded neurons was quantified by calculating the number of spikes in response to incremental (10–140 pA, 10 pA increment, 500 ms) depolarizing current injections (input-output relationship, I-O). I-O relationships were fit linearly, and the slope of the fitted line was defined as neuronal gain. For analysis of AP shape, a single AP was evoked by a 2 ms current step of minimal amplitude, sufficient to evoke an AP. Passive membrane properties of NI neurons and sag amplitude were calculated based on voltage responses to hyperpolarizing current pulses (–20 pA, 500 ms and –110 pA, 500 ms, respectively). Sag amplitude was calculated as the difference between the lowest reached amplitude during hyperpolarizing pulses (–100 pA, 500 ms) and steady-state current (voltage response during the last 50 ms of the pulse).

Voltage-clamp recordings to investigate the synaptic input to NI neurons were performed in standard ACSF at a holding potential of –50 mV. Since for the solutions used in the present study the calculated reversal potential for Cl^–^ currents was –90.5 mV, outward events represented inhibitory, and inward events represented excitatory postsynaptic currents (i/ePSCs), respectively.

For postsynaptic current analysis, a 200 s epoch of the voltage-clamp recoding was analyzed using Mini Analysis software (Synaptosoft Inc., Fort Lee, NJ, United States). Events were manually detected to measure frequency, amplitude and rise time of postsynaptic currents (obtained from single events) and a decay time constant (obtained from mean events).

In order to determine the pattern of spontaneous neuronal activity of NI neurons as well as their resting membrane potential, current-clamp recordings under a current zero protocol were performed, during which the first 60 s was used to calculate the nominated parameters. Only neurons characterized by low enough firing frequency to reliably assess the resting membrane potential, were included in the analysis. Recordings were analyzed using Spike 2 software running a custom script (CED). According to published criteria (Feng and Brown, 1999), neurons generating action potentials with the coefficient of variation (CV) of interspike intervals (ISI) below 0.5 were classified as regular firing, and neurons with CV of ISI above 0.5 were classified as irregular firing.

#### Post-recording Immunostaining

After recording, slices underwent immunofluorescent staining, in order to determine the neurochemical content of examined neurons. Slices were fixed overnight in 4% formaldehyde at 4°C. Fixed, free-floating sections were blocked and permeabilized with 10% NDS and 0.6% Triton X-100 in PBS, respectively, at 4°C overnight or for 3 h at RT. Subsequently, after washing in PBS, sections were incubated with a primary antibody solution: mouse anti-RLN3 (1:15, Supplementary Table 1), rabbit anti-proCCK (1:200, Supplementary Table 1), ExtrAvidin^®^-Cy3 (1:200, Sigma-Aldrich), 2% NDS and 0.3% Triton X-100 (Sigma-Aldrich) in PBS for 48-72 h at 4°C and, after several washing steps (in PBS), with secondary antibody solution: anti-mouse Alexa 647 (1:400) or anti-mouse Alexa 488 (1:400), anti-rabbit Alexa 647 (1:400), and 2% NDS in PBS at 4°C overnight. Slices were mounted onto glass slides, coverslipped with Fluoroshield and imaged using a fluorescence microscope (Axio Imager M2, Zeiss, with an A-Plan 10×/0.25 or EC Plan Neofluar 20×/0.25 objective). A lack of RLN3 or CCK immunoreactivity was not used as a prerequisite for assigning an NI neuron as non-RLN3 or non-CCK, due to possible dilution of antigen by the intrapipette solution during patch-clamp recording.

### Dendritic Tracing and Morphological Analysis

In studies to compare the dendritic morphology of type I (*n* = 24) and II (*n* = 14) NI neurons, microscopic images of whole biocytin-filled cells, acquired using an EC Plan Neofluar 20×/0.50 M27 objective (scaling: 0.36 μm in x and y, and 1.50 μm in z axis), were used for dendritic tracing and further 3D Sholl analysis (10 μm steps; Simple Neurite Tracer plugin in ImageJ; Longair et al., 2011), performed by an experimenter blinded to the cell type. Only well-filled neurons with clearly visible dendritic trees and no major truncations were imaged and analyzed. Tracing files were additionally processed using L-Measure software (Scorcioni et al., 2008), to acquire topological parameters, such as total dendritic length, maximal branch order, the number of primary dendrites, branches, bifurcations, and dendritic tips.

### Statistical Analysis

Statistical analysis was performed using GraphPad Prism V6.00 for Windows (GraphPad Software Inc., La Jolla, CA, United States). All data underwent outlier detection (ROUT method, Q = 1%) and outliers were eliminated from the analysis. Differences were considered statistically significant at *p* < 0.05. All tests were two-tailed. When data followed a normal distribution, unpaired *t*-tests or two-way ANOVA (RM in case of Sholl analysis) were used where appropriate, otherwise non-parametric Mann–Whitney or Kruskal–Wallis tests were used, as stated in the Results and figure descriptions. All values are provided as mean ± SD (for normally distributed data) or median with interquartile range (for data that did not pass normality tests).

## Results

### Medial Septum-Projecting Nucleus Incertus Neurons are Mostly Glutamatergic

RNAscope HiPlex *in situ* hybridization with probes for mCherry, RLN3, vesicular GABA (vGAT1) and glutamate (vGlut2) transporter mRNA, as well as corticotropin-releasing hormone receptor-1 (CRHR1) mRNA, was performed on brain sections from three rats with an intra-MS injection of retrograde pAAV-hSyn-mCherry viral vector, to determine the expression and distribution of different mRNA species in NI neurons that directly innervate the MS.

**FIGURE 1:**
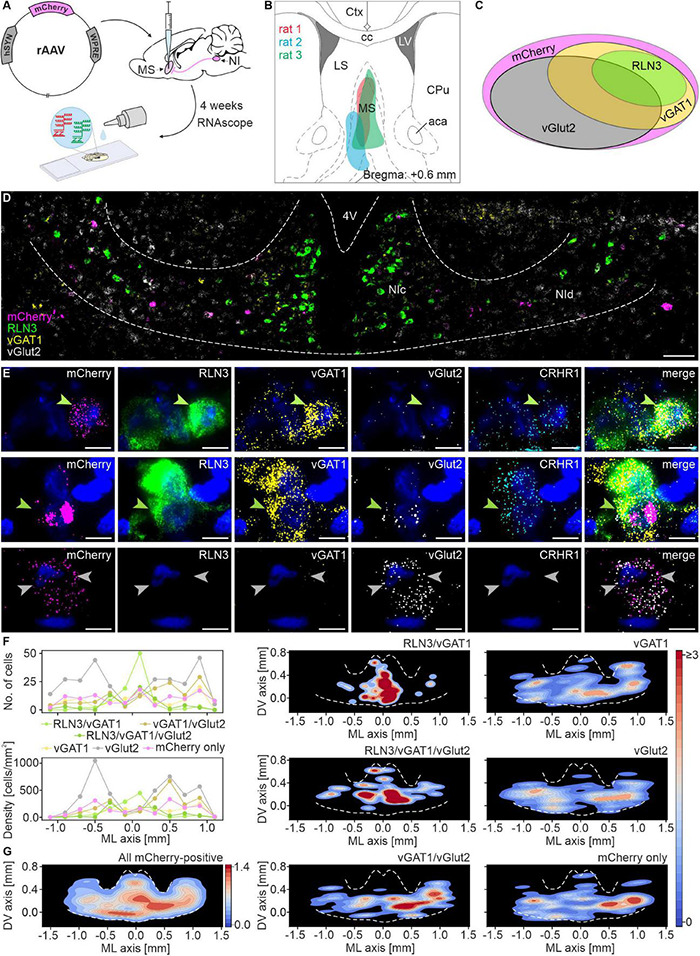
Multiple mRNA species detected in nucleus incertus neurons that directly innervate the medial septum. **(A)** Experimental procedure. **(B)** Reconstruction of the sites of retrograde pAAV-hSyn-mCherry viral vector injections in the MS. **(C)** Schematic of the proportions of and relationship between the distinguished types of MS-innervating NI neurons (area of each ellipse matches the percentage of each specific cell type). **(D)** Representative image of mCherry (pink), RLN3 (green), vGAT1 (yellow), and vGlut2 (light-gray) mRNA-expressing neurons in NI. Scale bar: 100 μm. **(E)** A series of images illustrating exemplary MS-innervating NI cells expressing each mRNA examined with DAPI-stained nuclei (blue): top, a RLN3/vGAT1/CRHR1 mRNA-expressing neuron (green arrowhead); middle, a RLN3/vGAT1/vGlut2/CRHR1 mRNA-expressing neuron (green arrowhead); bottom, two vGlut2 mRNA-expressing neurons (light gray arrowheads). Scale bars: 10 μm. **(F)** Medial-lateral axis distribution of the number (upper panel) and density (lower panel) of MS-innervating NI neurons. Bin size: 200 μm. **(G)** Density scatter plots with color-coded probability density function of all MS-innervating NI neurons, created for all (left panel) and for each individual type of MS-innervating NI neuron (middle and right panel); dotted white line delineates NI area. 4V, 4th ventricle; NIc, nucleus incertus *pars compacta*; NId, nucleus incertus *pars dissipata*; RLN3, relaxin-3; vGAT1, vesicular γ-aminobutyric acid (GABA) transporter; vGlut2, vesicular glutamate transporter.

Cell counting revealed that 38% of all mCherry mRNA-expressing neurons, were mCherry/vGlut2 mRNA-positive, and were located mostly in lateral parts of NI (NId). Twenty percent of NI neurons innervating the MS were identified as mCherry/RLN3 mRNA-positive (19% of total mCherry mRNA-expressing cells) and were located mainly in the medial NI area (containing NIc), and comprised two subgroups: (i) mCherry/RLN3/vGAT1 mRNA-expressing (60% of all mCherry/RLN3 mRNA-positive neurons), (ii) mCherry/RLN3/vGAT1/vGlut2-expressing (40% of all mCherry/RLN3 mRNA-positive neurons). Other cell types identified were: mCherry/vGAT1/vGlut2 mRNA-positive (17% of all mCherry mRNA-expressing cells), localized mainly in NId; mCherry/vGAT1 mRNA-expressing (12% of all mCherry mRNA-expressing cells, evenly distributed throughout NI); and finally, mCherry mRNA only (13% of all mCherry mRNA-expressing cells), also evenly distributed throughout the NI. In total, of all identified neuron types, those expressing vGlut2 mRNA (alone or in combination with other mRNA) accounted for 63% of NI neurons expressing mCherry (Figure 1 and Table 1).

**TABLE 1:** Combinations of mRNA species detected in nucleus incertus neurons innervating medial septum.

mRNA combination	Mean ± SD (%)	+ CRHR1 mRNA (%)
mCherry only	36 ± 18 (13)	6 ± 5 (16)
mCherry/vGAT1	32 ± 6 (12)	12 ± 4 (38)
mCherry/vGlut2	102 ± 13 (38)	26 ± 16 (25)
mCherry/vGAT1/vGlut2	47 ± 21 (17)	21 ± 12 (44)
mCherry/RLN3/vGAT1	31 ± 18 (12)	30 ± 16 (96)
mCherry/RLN3/vGAT1/vGlut2	21 ± 18 (8)	14 ± 13 (67)
All	269 ± 71 (100)	109 ± 56 (40)

Populations of CRHR1 mRNA-expressing neurons were present in all these groups, and these transcripts were particularly abundant in RLN3 mRNA-positive cells (96% of the mCherry/RLN3/vGAT1 population, and 67% of the mCherry/RLN3/vGAT1/vGlut2 cells) (Figure 1 and Table 1).

### Medial Septum Is Innervated by Different Populations of Peptidergic Nucleus Incertus Neurons

**FIGURE 2:**
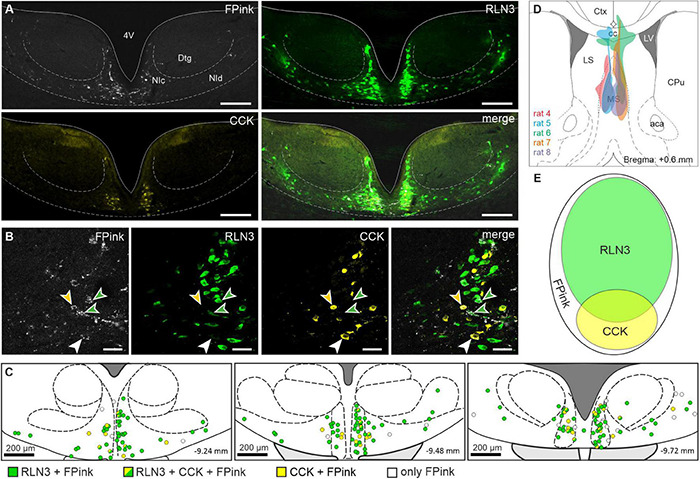
Neuropeptide content of nucleus incertus neurons innervating the medial septum. **(A)** Coronal sections through the NI illustrating the retrogradely-labeled neurons (FPink; white), RLN3 + (green), and CCK + (yellow) cells and a merged image illustrating the overlap of RLN3 and CCK in neurons that innervate MS. Scale bar: 200 μm. **(B)** Colocalization of FluoroPink with RLN3 (green arrowhead), CCK (yellow arrowhead), and both neuropeptides (white arrowhead). Scale bar: 50 μm. **(C)** Coronal sections illustrating the location of retrogradely-labeled cells within NI. A single circle represents one retrogradely-labeled neuron expressing RLN3 (green), CCK (yellow), both (half green—half yellow), or none of the neuropeptides (white). Scale bar: 200 μm. **(D)** Schematic illustrating the distribution of the FluoroPink retrograde tracer injection sites into MS. **(E)** Schematic of the proportions of and relationship between the distinguished types of MS-innervating NI neurons (area of each ellipse matches the percentage of each specific cell type). 4V, 4th ventricle; aca, anterior commissure, anterior part; cc, corpus callosum; CCK, cholecystokinin; Ctx, cerebral cortex; CPu, caudate putamen; DTg, dorsal tegmental nucleus; FPink, FluoroPink; LS, lateral septal nucleus; LV, lateral ventricle; MS, medial septum; NIc, nucleus incertus *pars compacta*; NId, nucleus incertus *pars dissipata*; RLN3, relaxin-3.

In studies to determine the neuropeptide content of NI neurons innervating MS, we performed an injection of the retrograde tracer, FluoroPink, into the MS of adult rats and subsequent immunofluorescent detection of RLN3 and CCK in the NI. Neurons containing FluoroPink were detected in all sections containing the NI (20.95 ± 4.6 neurons/section; 9–11 sections from 5 rats), with the highest density in the central part of the structure. The majority (∼62%) of all retrogradely-labeled neurons were RLN3-immunoreactive (13.04 ± 4.27 RLN3 neurons/section of 20.95 ± 4.6 FluoroPink neurons/section). At the same time, CCK-immunoreactive cells accounted for only 14% of the total neurons containing FluoroPink (2.85 ± 1.43 CCK neurons). Of the total retrogradely-labeled neurons, only a small proportion (∼7%) expressed both neuropeptides (1.45 ± 0.55 RLN3/CCK neurons/section). The remaining retrogradely-labeled neurons (17%) did not contain RLN3 or CCK (3.6 ± 0.63 neurons/section; Figure 2).

Reconstructions of the injection sites revealed that the spread of FluoroPink was similar in all rats and limited to the medial part of MS (Figure 2D), with injections slightly shifted toward one hemisphere. However, the retrogradely-labeled neurons were observed bilaterally, with more cells routinely observed ipsilaterally.

### Relaxin-3 and Cholecystokinin Nucleus Incertus Populations are Distinct

**FIGURE 3:**
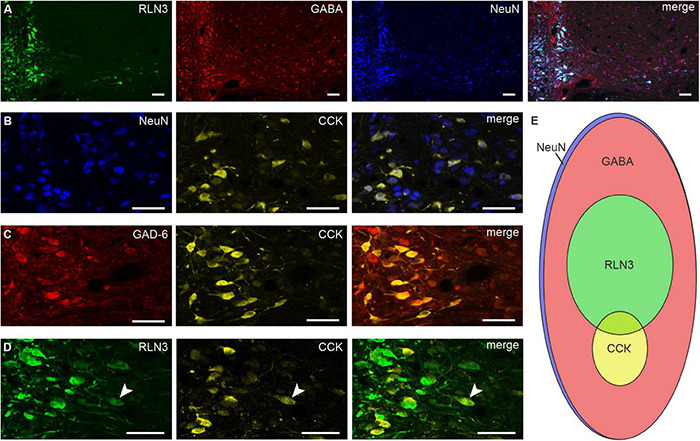
RLN3, CCK, and GABA neurons in the nucleus incertus. Representative images of single coronal sections at the level of the NI illustrating: **(A)** RLN3 + (green), GABA + (red), NeuN + (blue) neurons and merged image; **(B)** NeuN + (blue), CCK + (yellow) cells and merged image; **(C)** GAD-6 + (red), CCK + (yellow) neurons and merged image; **(D)** RLN3 + (green) and CCK + (yellow) neurons, and sparse peptide colocalization (white arrowhead). Scale bars: 50 μm. **(E)** Schematic of the proportions of and relationship between all, RLN3 +, CCK +, and GABAergic NI neurons (area of each ellipse matches the percentage of each specific cell type). CCK, cholecystokinin; GABA, γ-aminobutyric acid; GAD-6, glutamic acid decarboxylase 65/67; NeuN, neuronal nuclear protein; RLN3, relaxin-3.

In experiments to confirm data describing the NI as a GABAergic nucleus (Ma et al., 2007), the number of neurons positive for GABA, RLN3, and NeuN was quantified. A majority of neurons in the NI were GABAergic (∼96%; 306.90 ± 10.02 GABA–NeuN of 318.60 ± 14.49 NeuN neurons/section), of which ∼29% were also positive for RLN3 (92.25 ± 6.60 RLN3–NeuN of 318.60 ± 14.49 NeuN neurons; Figure 3A). In order to identify the classical neurotransmitter content of the NI CCK neurons, an immunofluorescent staining against CCK and NeuN or GAD-6 was conducted. Most, if not all, CCK neurons were GABAergic, and accounted for only ∼8% of total NI neurons (25.89 ± 1.48 CCK–NeuN of 333.39 ± 13.19 NeuN neurons; Figures 3B,C). Immunofluorescent detection of RLN3 and CCK revealed that these neuropeptide neurons are largely separate populations, although a small number (∼4%) of RLN3 neurons appeared positive for CCK (3.98 ± 0.60 neurons/section) (Figure 3D). CCK neurons were much fewer in number and more confined to the midline NIc than those containing RLN3, and quantification revealed that the CCK population (25.89 ± 1.48 neurons/section) was approximately a third the size of the RLN3 neurons (92.25 ± 6.60 neurons/section) (Figure 3E). Immunofluorescence for CCK was confined to the soma, with no labeled proximal processes visible.

### Relaxin-3 and Cholecystokinin Neuron Populations Display Diverse Colocalization With the Calcium-Binding Proteins, Calbindin and Calretinin

**FIGURE 4:**
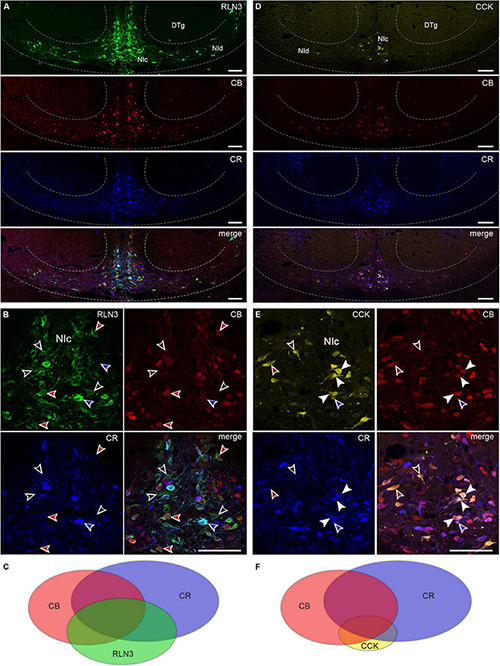
RLN3 and CCK colocalization with calcium-binding proteins in nucleus incertus neurons. **(A)** Overview of immunofluorescence in the NI region for RLN3 (green), CB (red), CR (blue), and a merged image (bottom); and **(B)** combinations of colocalization of the tested markers: RLN3-CB neurons devoid of CR (red arrowheads), RLN3-CR neurons devoid of CB (blue arrowheads), RLN3 neurons devoid of CR, and CB (black arrowheads). **(C)** Schematic of the proportions of and relationships between RLN3 and the tested calcium-binding proteins. **(D)** Representative images of coronal sections through the NI with immunofluorescence for CCK (yellow), CB (red), and CR (blue), and a merged image illustrating colocalization of the tested markers. **(E)** Colocalization of CCK with calcium-binding proteins: CCK neurons positive for CB and CR (white arrowheads), CCK neurons positive for only CB (green arrowheads), or CR (blue arrowheads), or neither calcium-binding protein (black arrowheads). **(F)** Schematic of the relationship between CCK, CB, and CR. Scale bars: 100 μm. CB, calbindin; CCK, cholecystokinin; CR, calretinin; DTg, dorsal tegmental nucleus; NIc, nucleus incertus *pars compacta*; NId, nucleus incertus *pars dissipata*; RLN3, relaxin-3.

In order to assess the relative populations of NI neurons expressing calcium-binding proteins in relation to their RLN3 and CCK content, a series of immunohistochemical studies was performed. Both calbindin- and calretinin-immunoreactivity were mainly confined to the soma and within proximal processes. RLN3, calbindin and calretinin were detected in all possible colocalization combinations within NI neurons (Figures 4A–C), producing a total of seven different types of cells including single-, double-, and triple-labeled neurons (Supplementary Table 3). Quantification of these cell types revealed that ∼18% of total neurons detected were labeled for all three markers (32.46 ± 3.66 of 176.0 ± 11.87 neurons), as well as a relatively even spread of pairwise colocalization (Supplementary Table 3). Of the total neurons detected, ∼7% were positive for RLN3 and calbindin, whereby calbindin was detected in ∼56% of RLN3 neurons, and RLN3 was detected in ∼48% of total calbindin neurons. For calretinin, ∼11% of the total neurons detected were positive for RLN3 and calretinin, whereby calretinin was detected in ∼65% of RLN3 neurons, and RLN3 was present in ∼41% of calretinin neurons. Of the total neurons detected, ∼14% of neurons exhibited calbindin and calretinin colocalization in the absence of RLN3, in addition to single-label immunostaining for RLN3 (∼9%), calbindin (∼13%), and calretinin (∼28%). Within those neurons positive for the calcium-binding proteins, but not RLN3, ∼45% of calretinin neurons were also positive for calbindin and ∼61% of calbindin neurons were positive for calretinin.

Next, we assessed whether CCK neurons expressed a particular calcium-binding protein. Similar to RLN3 neurons, we observed all possible colocalization combinations of calbindin and calretinin with CCK in NI neurons (Supplementary Table 4 and Figures 4D–F). Quantification of these cell types revealed that ∼9% of neurons detected were labeled for all three markers (15.50 ± 1.61 of 169.67 ± 8.59 neurons/section). Of the total neurons detected, ∼3% were positive for CCK and calbindin, whereby calbindin was detected in ∼64% of CCK neurons, and CCK was present in ∼20% of total calbindin neurons. For calretinin, ∼18% of the total neurons detected were positive for CCK and calretinin, whereby calretinin was detected in ∼58% of CCK neurons, and CCK was detected in ∼18% of calretinin neurons. Of the total neurons detected, ∼18% of neurons exhibited calbindin and calretinin colocalization in the absence of CCK, in addition to single-label staining for CCK (∼6%), calbindin (∼31%), and calretinin (∼31%). Within the neurons positive for the calcium-binding proteins, but not CCK, ∼45% of calretinin neurons were positive for calbindin and ∼43% of calbindin neurons were calretinin-positive.

### Medial Septum Is Predominantly Innervated by Type I Nucleus Incertus Neurons

**FIGURE 5:**
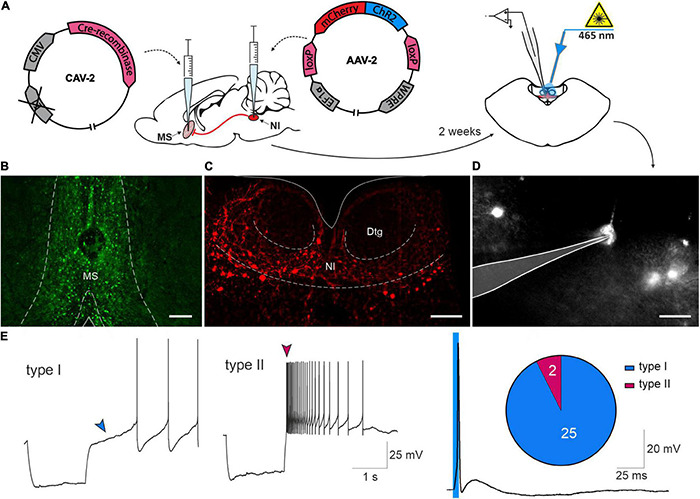
Electrophysiological characterization of nucleus incertus neurons innervating the medial septum. **(A)** Experimental procedure. **(B)** Representative image of an MS injection site, illustrating Cre recombinase expression (green). Scale bar: 200 μm. **(C)** Representative image of NI illustrating mCherry protein expression in the NI region (red). Scale bar: 200 μm. **(D)** Image of NI neuron expressing mCherry protein recorded *ex vivo*, with the recording pipette outlined. Scale bar: 30 μm. **(E)** Representative traces of a current clamp recording demonstrating the delayed return to the baseline activity (blue arrowhead), characteristic of type I NI neurons (**left**) and the characteristic rebound depolarization (dark pink arrowhead) of type II NI neurons (**middle**), in response to a hyperpolarizing current pulse. Action potential recorded in NI neuron expressing mCherry, evoked in response to a light pulse (465 nm, 5 ms, blue line) (**right**), and pie chart of the number of type I (blue) and type II (dark pink) neurons innervating MS. MS, medial septum; NI, nucleus incertus.

Electrophysiological properties of NI neurons innervating MS, were characterized in whole-cell patch-clamp recordings performed using NI-containing coronal brain slices from rats that received an intra-MS injection of a CAV2-CMV-Cre viral vector and intra-NI injections of an AAV2-EF1a-DIO-ChR2(H134R)-mCherry viral vector (Figures 5A–C). Recordings were performed from NI neurons that expressed mCherry fluorescent protein and responded to blue light (λ = 465 nm) stimulation with light-evoked action potentials (Figures 5D,E), thereby characterized as directly innervating the MS. In mCherry expressing NI neurons every single light pulse from a series of at least 20 pulses (5 ms, 0,1 Hz) evoked a single action potential. Recordings were performed in the presence of ionotropic glutamate and GABA receptor antagonists (CNQX and DL-AP5, and gabazine, respectively), to further ensure that only neurons directly innervating the MS were recorded. Of 27 mCherry-expressing NI neurons directly sensitive to photostimulation, 25 (93%) were defined as type I neurons, characterized by a marked delay in the return of the membrane potential to baseline levels at the offset of a hyperpolarizing current pulse (Blasiak et al., 2015). Only 2 of 27 (7%) NI neurons directly innervating MS, were defined as type II neurons, characterized by a rebound depolarization after the hyperpolarizing current pulse (Figure 5E).

### Type I and II Nucleus Incertus Neurons Have Different Intrinsic Membrane Properties

In studies to further characterize type I and type II NI neurons, whole cell, current-clamp recordings were performed in brain slices from naïve rats. Overall, 291 NI neurons were recorded, of which 191 were characterized as type I and 100 as type II NI neurons.

**FIGURE 6:**
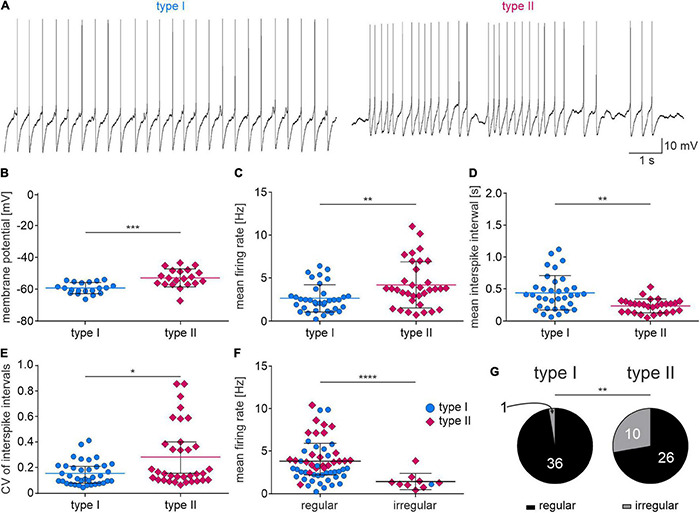
Spontaneous activity of type I and II NI neurons. **(A)** Exemplary zero current-clamp trace recording of type I (**left**) and type II (**right**) NI neurons’ spontaneous activity. **(B–F)** Strip charts representing differences in active properties between type I and II NI neurons. Lines and whiskers represent mean and SDs **(B,D)**, or median and interquartile range **(C,E,F)**. *(*p* < 0.1), **(*p* < 0.01), ***(*p* < 0.001), ****(*p* < 0.0001), unpaired *t*-test in **(B,D)**, unpaired Mann-Whitney test in **(C,E,F)**. **(G)** Proportions of regular and irregular firing type I NI neurons (**left**) and type II NI neurons (**right**), **(*p* = 0.0031, Fisher’s exact test).

Type I NI neurons had a significantly lower resting membrane potential than type II neurons (Figures 6A,B and Table 2), and a lower mean firing rate (Figure 6C and Table 2). Consequently, the mean interspike interval (ISI) in type I neurons was significantly longer than in type II NI neurons (Figure 6D and Table 2). Comparison of the coefficient of variation (CV) of the ISI revealed that in type I neurons, the CV of ISI was significantly smaller than in type II neurons (Figure 6E and Table 2). According to published criteria (Feng and Brown, 1999), neurons with a CV of ISI below 0.5 were characterized as regular firing, and neurons with a CV of ISI above 0.5 were characterized as irregular firing. Comparison of the mean firing rate of regular and irregular NI neurons revealed that irregular neurons had a significantly lower firing rate than regular neurons (Figure 6F and Table 2). Interestingly, among the irregular NI neurons, only one was type I, and the remaining type I neurons were characterized by regular spiking. Fisher’s exact test revealed a significant difference between the proportion of irregular and regular neurons among type I and II NI neurons (Figure 6G and Table 2), with a higher percentage of regular type I NI neurons (97%), than type II NI neurons (72%, Figure 6G).

**TABLE 2:** Firing and action potential properties of type I and II nucleus incertus neurons.

Parameter	Type I (n)	Type II (n)			Statistics
**Firing properties**					
Spontaneous firing rate (Hz)	2.38 ± 1.64 (36)	3.70 ± 3.944 (34)			^†^ ***p* = 0.006**
Interspike interval (s)	0.44 ± 0.27 (35)	0.24 ± 0.11 (28)			^#^ ***p* = 0.0002**
CV of interspike interval	0.12 ± 0.14 (37)	0.15 ± 0.28 (34)			^†^ ***p* = 0.028**
Threshold (mV)	−43.98 ± 7.32 (130)	−48.09 ± 3.95 (78)			^†^ ***p* < 0.0001**
Rheobase (nA)	0.13 ± 0.05 (110)	0.06 ± 0.01 (52)			^§^ ***p* < 0.0001**
Time to first AP (ms)	152.9 ± 127.8 (191)	18.79 ± 7 (100)			^†^ ***p* < 0.0001**
Neuronal gain	0.08 ± 0.003 (123)	0.14 ± 0.005 (69)			Comparison of slopes: *F* = 129.806 DFn = 1 DFd = 2337, ***p* < 0.0001**
Regular firing	36/37	26/36			Fisher’s exact test, *p* = 0.0031

**Membrane properties**					

Resting membrane potential (mV)	−59.04 ± 3.52 (20)	−52.64 ± 5.757 (20)			^#^ ***p* = 0.0002**
Resistance (MOhm)	791.9 ± 426.5 (96)	789.1 ± 391.3 (53)			^†^*p* = 0.618
Time constant (ms)	56.38 ± 29.18 (96)	40.51 ± 31.18 (53)			^†^ ***p* = 0.0002**
Capacity (pF)	72.07 ± 29.6 (94)	57.58 ± 21.61 (53)			^†^ ***p* < 0.0001**
Sag amplitude (mV)	−3.97 ± 3.72 (32)	−2.52 ± 2.40 (17)			^†^ ***p* = 0.038**

**Single AP properties**					

Threshold (mV)	−39.18 ± 5.25 (48)	−39.6 ± 5.44 (38)			^§^ *p* = 0.714
10–90 rise (us)	295.6 ± 62.2 (48)	301.8 ± 89.8 (38)			^†^*p* = 0.617
Amplitude (mV)	55.37 ± 19.57 (48)	51.92 ± 15.94 (38)			^†^ ***p* = 0.02**
Half-width (us)	699.2 ± 104.1 (48)	654.7 ± 137.8 (38)			^§^ *p* = 0.092

**AHP properties**					

	**medium AHP**	**fast AHP**	**medium AHP**	**m AHP only**	

AHP trough (mV)	−88.0 ± 2.73 (48)	−65.0 ± 2.9 (18)	−77.1 ± 1.96 (17)	−79.02 ± 4.26 (20)	One way ANOVA: ***p* < 0.0001** *F*_(3,99)_ = 266.9; Tukey’s multiple comparisons: I mAHP vs. II fAHP ***p* ≤ 0.0001** I mAHP vs. II mAHP ***p* ≤ 0.0001** I mAHP vs. II mAHP only ***p* ≤ 0.0001** II fAHP vs. II mAHP ***p* ≤ 0.0001** II fAHP vs. II mAHP only ***p* ≤ 0.0001** II mAHP vs. II mAHP only ns

	**Medium AHP**	**Fast AHP**	**Medium AHP**	**m AHP only**	

Peak—AHP time (ms)	9.136 ± 3.08 (45)	2.51 ± 1.48 (18)	65.7 ± 42.93 (18)	42.69 ± 34.4 (20)	Kruskal—Wallis test: ***p* < 0.0001;** Dunn’s multiple comparisons: I mAHP vs. II fAHP ***p* ≤ 0.001** I mAHP vs. II mAHP ***p* ≤ 0.0001** I mAHP vs. II mAHP only ***p* ≤ 0.0001** II fAHP vs. II mAHP ***p* ≤ 0.0001** II fAHP vs. II mAHP only ***p* ≤ 0.0001** II mAHP vs. II mAHP only ns

*Bold values denote statistical significance at the p < 0.05 level. ^†^Unpaired Mann–Whitney test; ^#^Unpaired t-test with Welch’s correction; ^§^Unpaired t-test.*

**FIGURE 7:**
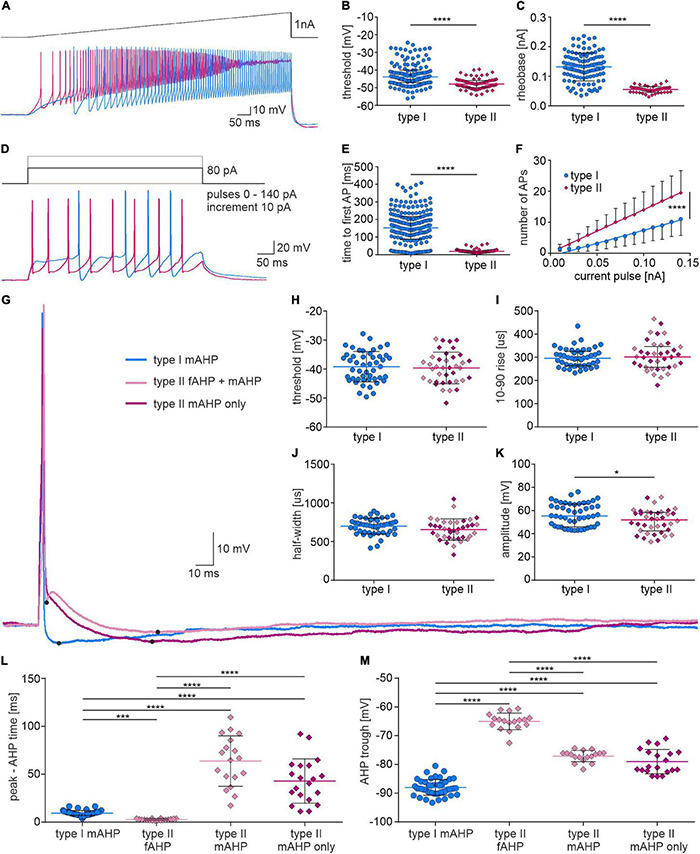
Firing properties and single action potential shape of type I and II NI neurons. **(A)** Current clamp protocol (**upper trace**) and voltage response (**lower trace**) of type I (blue) and type II (pink) NI neurons to applied current. **(B)** Action potential threshold, and **(C)** rheobase calculated from voltage responses shown in **(A)**, ****[*p* < 0.0001, unpaired Mann-Whitney test in **(B)**, unpaired *t*-test, in **(C)**]. **(D)** Current clamp protocol (upper trace) and voltage response of type I (blue) and type II (pink) NI neurons to depolarizing current pulse (+80 pA). **(E)** Time to first AP (*p* < 0.0001, unpaired Mann-Whitney test), and **(F)** number of action potentials of type I and type II NI neurons vs. the intensity of injected current, calculated from the voltage responses shown in **(D)**. The slopes of the regression lines fitted to the experimental data represents the gain, which differed significantly between groups, ****(*p* < 0.0001, comparison of regression lines slopes). **(G)** Action potentials of type I and II NI neurons evoked by a single depolarizing current pulse, and **(H–M)** their properties. Note two different AP waveforms were recorded from type II NI neurons (with single mAHP and with fAHP and mAHP). *(*p* = 0.02, unpaired Mann-Whitney test). **(L)** AP peak to AHP trough time, and **(M)** AHP trough in APs of type I neurons (type I mAHP), in APs of type II neurons with fAHP and mAHP (type II fAHP and type II mAHP) and in APs of type II neurons with single mAHP (type II mAHP), ***(*p* ≤ 0.001), ****(*p* ≤ 0.0001), Kruskal-Wallis test in **(L)** and one-way ANOVA in **(M)**. AHP, afterhyperpolarization; AP, action potential; fAHP, fast afterhyperpolarization; mAHP, medium afterhyperpolarization.

In order to further test the intrinsic properties of type I and II NI neurons, their action potential threshold, rheobase and excitability were compared. Analysis revealed that type II neurons had a more hyperpolarized threshold for AP generation, a lower rheobase and a shorter time to first spike (Figures 7A–E and Table 2). Moreover, type II neurons were more excitable than type I, as neuronal gain, represented by the slopes of the straight regression lines fitted to the experimental data, had a significantly higher value for type II than in type I cells (Figure 7F and Table 2).

To assess possible differences in action potential characteristics between type I and II NI neurons, the shape of a single action potential evoked by a depolarizing current pulse was compared. Analysis of type I and II NI neuron AP parameters revealed that in the AP of type I neurons only medium after hyperpolarization (type I mAHP, Figures 7G–M) was present, whereas in the AP of type II NI neurons, either single medium AHP (type II mAHP only, 20 of 38 type II neurons, 52.63%) or fast (type II fAHP) and medium AHP (type II mAHP), separated by brief ADP (18 of 38 type II neurons, 47.37%) was recorded. The time from AP peak to AHP trough differed significantly between types of AHPs observed in type I and type II NI neurons. Time from peak to AHP trough in type I neuron APs was longer than the time to type II fAHP, but shorter than the time to type II mAHP (Figure 7L and Table 2). Moreover, in the AP of type I neurons, the AHP trough reached more hyperpolarized values than troughs of both fAHP and mAHP of type II neurons AP (Figure 7M and Table 2).

**FIGURE 8:**
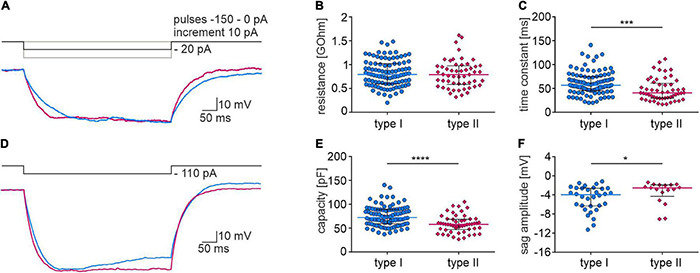
Membrane properties of type I and II NI neurons. **(A)** Current clamp protocol (**upper trace**) and voltage response (**lower trace**) of type I (blue) and type II (pink) NI neurons to applied hyperpolarizing current pulse (–20 pA). **(B)** Membrane resistance, and **(C)** time constant of type I and type II NI neurons, *** (*p* = 0.0002, unpaired Mann-Whitney test). **(D)** Current clamp protocol (**upper trace**) and voltage response (**lower trace**) of type I (blue) and type II (pink) NI neurons to applied hyperpolarizing current pulse (–110 pA). **(E)** Membrane capacity of type I and type II neurons calculated based on the resistance and time constant measured from voltage response to –20 pA current pulse, ****(*p* < 0.0001, unpaired Mann-Whitney test). **(F)** Voltage sag amplitude of type I and II neurons calculated from the voltage response to the current protocol shown in **(D)**, *(*p* = 0.038, unpaired Mann-Whitney test).

Based on a voltage response to a hyperpolarizing current pulse, passive membrane properties and the voltage sag amplitude of type I and type II neurons were calculated. Analysis revealed that the time constant and capacitance of type II neurons had lower values than type I neurons, whereas the membrane resistance did not differ between the examined types of NI neurons (Figures 8A–C,E and Table 2). The sag amplitude in type I neurons was significantly larger than in type II NI neurons (Figures 8D,F and Table 2).

### Type I and Type II Neurons Receive Different Synaptic Inputs

Possible differences in type I and type II NI neuron synaptic inputs were assessed by analyzing parameters of spontaneous postsynaptic currents. Type I (*n* = 26) and type II (*n* = 19) neurons were recorded in voltage-clamp mode (holding potential –50 mV) and inhibitory and excitatory postsynaptic currents (iPSCs and ePSCs, respectively) were manually detected to measure frequency, amplitude and rise time of postsynaptic currents and decay time constant of the averaged current trace.

**FIGURE 9:**
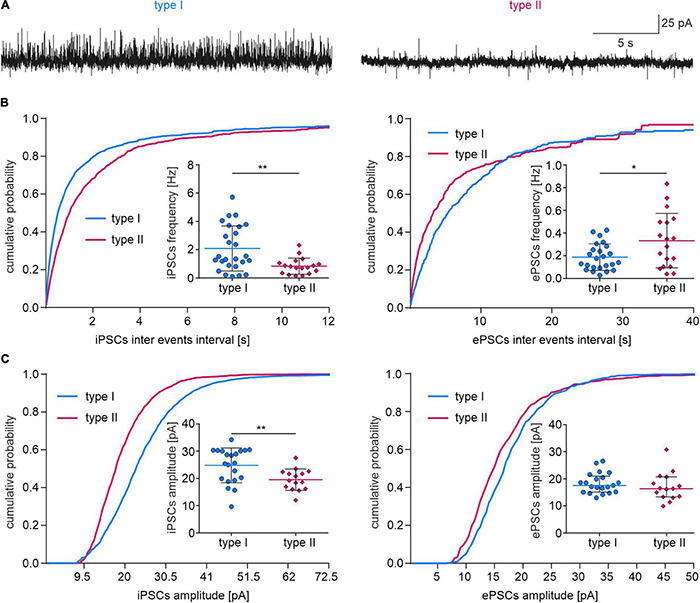
Synaptic inputs to type I and II NI neurons. **(A)** Exemplary traces of voltage clamp recordings (holding potential –50 mV) illustrating spontaneous inhibitory (upward deflections) and excitatory (downward deflections) postsynaptic currents in type I and type II NI neurons. **(B)** Cumulative frequency distribution histograms of the inhibitory (**left**) and excitatory (**right**) PSC inter-event intervals (bin size = 20 ms). **Insets**, comparisons of PSCs frequencies *(*p* = 0.014), **(*p* = 0.003), unpaired *t*-tests. **(C)** Cumulative frequency distribution histograms of sIPSC and sEPSC amplitudes (bin = 1 pA). **Insets**, comparisons of PSC amplitudes, **(*p* = 0.008, unpaired *t*-test). ePSC, excitatory postsynaptic current; iPSC, inhibitory postsynaptic current.

A higher iPSC frequency and amplitude, and lower ePSC frequency was recorded in type I than in type II NI neurons (Figures 9A–C and Table 3), indicating a stronger synaptic inhibition of type I than type II cells. The remaining postsynaptic current parameters, including rise time and tau in both iPSCs and ePSCs, and the amplitude of ePSCs, did not differ between the two neuronal types (Table 3).

**TABLE 3:** Parameters of inhibitory and excitatory postsynaptic currents in type I and II nucleus incertus neurons.

PSC parameters	iPSC		Statistics	ePSC		Statistics
	Type I (n)	Type II (n)		Type I (n)	Type II (n)	
Frequency (Hz)	2.08 ± 1.59 (26)	0.83 ± 0.57 (18)	^#^ ***p* = 0.003**	0.19 ± 0.12 (24)	0.33 ± 0.24 (18)	^#^ ***p* = 0.014**
Rise time (ms)	2.64 ± 0.6 (26)	2.63 ± 0.58 (19)	^#^*p* = 0.955	2.62 ± 0.62 (26)	2.46 ± 0.56 (19)	^#^*p* = 0.394
Amplitude (pA)	24.78 ± 6.37 (21)	19.54 ± 3.92 (15)	^#^ ***p* = 0.008**	17.52 ± 5.9 (21)	16.34 ± 7.38 (15)	^†^*p* = 0.21
Tau (ms)	4.37 ± 1.28 (26)	5 ± 1.26 (19)	^#^*p* = 0.107	2.97 ± 2.32 (26)	2.34 ± 0.88 (19)	^†^*p* = 0.087

*Bold values denote statistical significance at the p < 0.05 level. ^#^Unpaired t-test; ^†^Mann-Whitney unpaired t-test.*

### Relaxin-3- and Cholecystokinin-Positive Cells are Predominantly Type I Nucleus Incertus Neurons

**FIGURE 10:**
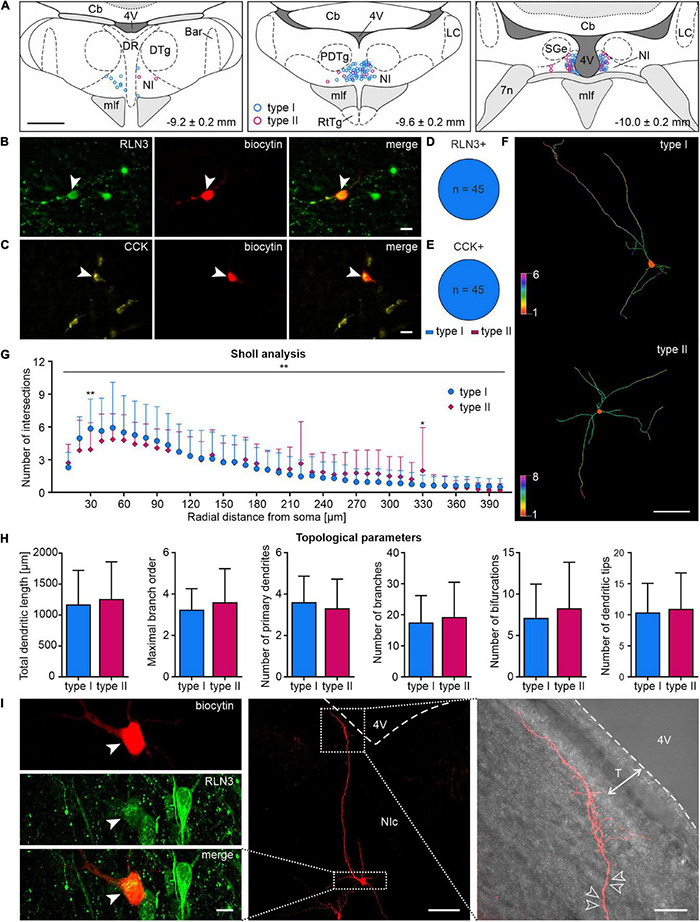
Localization, morphology and neuropeptide content of type I and II NI neurons. **(A)** Reconstruction (relative to bregma, lower right corner), of the localization of type I (blue circles) and type II (pink circles) NI neurons recorded during patch clamp experiments. **(B)** Post-recording immunofluorescent identification of NI neurons: image of RLN3+ NI neuron (green, white arrowhead); biocytin (red, white arrowhead) and merge. Scale bar: 20 μm. **(C)** Image of CCK+ NI neuron (yellow, white arrowhead); biocytin (red, white arrowhead) and merge. Scale bar: 20 μm. **(D,E)** Electrophysiological types of RLN3+ and CCK+ NI neurons, respectively. **(F)** 2D view of representative dendritic traces of type I (**top**) and type II (**bottom**) neurons, drawn with colors representing the number of dendrites intersecting with Sholl spheres. Scale bar: 100 μm. **(G)** Sholl analysis and comparison of the dendritic trees of type I and II NI neurons; despite a significant interaction between neuron type and distance from soma, significant differences, indicated by asterisks, were found only at 30 and 330 μm from soma. Significance indicated by two-way RM ANOVA and Uncorrected Fisher’s LSD *post-hoc* test: *p < 0.05, **p < 0.01. **(H)** Topological parameters of type I and II NI neurons (total dendritic length, maximal branch order, number of primary dendrites, branches, bifurcations and dendritic tips). **(I)** Images of a representative, biocytin-filled NI RLN3+ neuron (**left panel**, cell body indicated with a white arrowhead), with thin, tuft-like distal dendrites pointing toward the 4th ventricle (**right panel**; empty arrowheads indicate exemplary dendritic spines; white dotted line with arrows indicates the extent of tanycytes, T). Boxed areas in the **middle panel** represent the area surrounding the cell body (**left panel**) and the tuft-like distal dendrites (**right panel**). 4V, 4th ventricle; Bar, Barrington’s nucleus; Cb, cerebellum; CCK, cholecystokinin; DR, dorsal raphe nucleus; DTg, dorsal tegmental nucleus; mlf, medial longitudinal fasciculus; NI, nucleus incertus; NIc, nucleus incertus *pars compacta*; RLN3, relaxin-3; RtTg, reticulotegmental nucleus of the pons; T, tanycytes.

In order to determine any correlation between the biochemical and electrophysiological properties of NI neurons, after patch-clamp recordings, brain slices were immunostained for RLN3 (Figures 10A,B) and CCK (Figures 10A,C). The biochemical profile of 90 NI neurons was identified and all of RLN3-positive neurons were electrophysiological type I neurons (45/45, 100%, Figure 10D), in line with our previous report (Blasiak et al., 2015). Similarly, all CCK NI neurons identified were type I neurons (45/45, 100%, Figure 10E). In the overall post-recording immunostaining, one cell was positive for both RLN3 and CCK. This neuron was also type I, and was not included in the described cell counts.

### Type I and II Nucleus Incertus Neurons are Not Morphologically Distinct

Morphological features of type I and II NI neurons were analyzed to investigate any possible structural divergence. Two-way repeated measures ANOVA of Sholl analysis data revealed a significant interaction between the distance from soma and cell type, but a *post hoc* Uncorrected Fisher’s LSD test revealed only minor, local differences in dendritic tree complexity (Figures 10F,G and Supplementary Table 5). No significant differences were detected in the topological parameters (total dendritic length, maximal branch order, number of primary dendrites, branches, bifurcations, and dendritic tips) of type I and II NI neurons (Figure 10H and Supplementary Table 6).

Additionally, during the dendritic tracing studies, we observed that the cell bodies of both type I and II neurons varied in shape and size, with fusiform, stellate, round, and occasional pyramidal-like shapes detected. Axons, identified by the presence of an axonal bleb (Debanne et al., 2011; Hu and Shu, 2012), arose most frequently from first-order dendrites. Many neurons were spiny and/or possessed varicose dendrites, especially in the distal part of their dendritic tree, as described, for example, in the ventral tegmental area and basal forebrain (Phillipson, 1979; Brauer and Winkelmann, 1987; Brauer et al., 1988). Interestingly, in 34% (13/38) of the neurons examined, distal varicose dendrites tended to be tuft-like (very thin, extensively branched) and located in close proximity to, and pointing toward, the fourth ventricle (Figure 10I). The majority (69%, 9/13) was identified as type I neurons, of which 56% (5/9) was RLN3-immunoreactive, whereas 31% (4/13) was type II, with 25% (1/4) considered RLN3-immunopositive. Notably, eight dendritically-traced neurons were RLN3-immunoreactive, of which six (75%) possessed tuft-like, varicose dendrites.

## Discussion

These studies provide a comprehensive neurochemical and electrophysiological characterization of NI neurons innervating the MS, including detailed descriptions of the chemoarchitecture, morphology and electrophysiological features of type I/II NI neurons. Viral-based, retrograde neural tract-tracing combined with *in situ* hybridization revealed that the MS is innervated by RLN3/GABAergic, and putative glutamatergic (vGlut2 mRNA-expressing) NI neurons. Notably, we demonstrated that a population of NI neurons innervating the MS display a unique, dual neurochemical phenotype for GABAergic and glutamatergic neurotransmission. We also observed that 40% of MS-projecting NI neurons express CRHR1 mRNA. Using classical retrograde tracing and immunohistochemical methods, we observed that the MS is innervated by RLN3 and CCK-containing NI neurons that form largely separate neuronal populations belonging to a broader GABAergic neuronal pool expressing the calcium-binding proteins, calbindin and calretinin. Finally, using retrogradely transported AAV vectors and optogenetic tagging, we demonstrated that the MS is innervated almost exclusively by I_A_ expressing, type I NI neurons. Results of our single-cell electrophysiological studies revealed that type I and II NI neurons differ significantly in their passive and active membrane properties, action potential shape and neurochemical profile. Our results demonstrate that the rat NI–MS pathway is formed by distinctive, type I NI neurons, and suggest that the septohippocampal pathway is controlled by a discrete NI neuronal network with specific electrophysiological and neurochemical features.

The role of the NI in the control of hippocampal theta rhythm and related locomotor activity and spatial memory, has been established and reliably demonstrated in rats and mice (Nuñez et al., 2006; Cervera-Ferri et al., 2011; Ma et al., 2013; Farooq et al., 2016; Albert-Gascó et al., 2017; Haidar et al., 2017; Olucha-Bordonau et al., 2018; Szőnyi et al., 2019; Lu et al., 2020). Given that the dorsal hippocampus receives relatively sparse inputs from the NI (Goto et al., 2001; Olucha-Bordonau et al., 2003), the direct and dense projection from the NI to the MS, emerges as a critical pathway for NI control of hippocampal theta rhythm (Ma et al., 2009; Szőnyi et al., 2019). Results of our current multiplex *in situ* hybridization and immunohistochemical experiments confirmed previous reports that the MS is innervated by RLN3 NI neurons and that virtually all RLN3 neurons are GABAergic (Ma et al., 2007). Our current neural tract-tracing results differ quite markedly in the percentage of RLN3 neurons innervating the MS, depending on whether they were obtained using peptide or mRNA labeling. While FluoroPink based tract-tracing combined with immunohistochemical detection of RLN3 indicated that RLN3 cells constitute ∼62% of NI neurons innervating MS, viral-based tracing combined with *in situ* hybridization indicated that RLN3 mRNA is present in only 20% of NI neurons innervating MS. This discrepancy may be due to the use of colchicine in experiments with immunohistochemical staining, and the accumulation of RLN3 in a substantial number of neurons during the survival period. RLN3 is implicated in the control of circadian rhythms, and therefore a circadian variation in RLN3 mRNA and peptide levels is possible (Smith et al., 2012; Blasiak et al., 2013, 2017). Also, to some extent, the differential location of the injection sites for the viral vectors and FluoroPink, may have resulted in the labeling of different NI cell populations, as the retrograde AAVs was injected more ventrally in the MS than FluoroPink. In addition, the observed differences could be a consequence of non-uniform uptake of the viral vector across the presynaptic membranes of different neurons at the injection site (Yang et al., 2002; Davidson and Breakefield, 2003), as well as the sensitivity of the combined assays.

In the current study, we demonstrated that a substantial population of NI neurons innervating the MS express vGlut2 mRNA. Notably, most previous studies have focused on the importance of RLN3/GABA NI neurons in the NI–MS pathway, and on the involvement of the NI in theta rhythm control and related behaviors (Olucha-Bordonau et al., 2012; Ma et al., 2013; Albert-Gascó et al., 2018; Haidar et al., 2019; Szőnyi et al., 2019). In contrast, glutamatergic NI neurons and their projections were much less studied (Cervera-Ferri et al., 2012; Szőnyi et al., 2019; Lu et al., 2020), although Cervera-Ferri et al. (2012) reported that the MS is innervated by glutamatergic NI neurons in rats, and Lu et al. (2020) demonstrated, using vGlut2-Cre mice, that activation of NI glutamatergic cells significantly increased locomotion, arousal and hippocampal theta power. Earlier, Szőnyi et al. (2019) reported a lack of NI-originating fibers in the MS after injection of a Cre-dependent AAV driving expression of YFP into the NI of Vglut2-Cre mice. Here we demonstrated, using a comparable marker of glutamatergic neurons (vGlut2 mRNA), that NI glutamatergic neurons strongly innervate the MS in the rat, and that some of these neurons co-express RLN3 and/or vGAT1 mRNA. This discrepancy may be due to the targeting of the Cre-dependent AAVs to the mouse NIc by Szőnyi et al. (2019), as we have shown that vGlut2 mRNA positive neurons innervating MS are primarily located in the lateral NI (NId). Our *in situ* hybridization results indicate that this also applies to the general population of NI vGlut2 mRNA-expressing neurons. Moreover, we observed that the RLN3 mRNA-expressing MS-innervating neurons are present predominantly in the medial NI (containing NIc), while those co-expressing RLN3, vGAT1, and vGlut2 mRNA were located more laterally and were more dense in the NId than “RLN3/GABA only” neurons. In contrast, the distribution of MS-innervating, “purely” GABAergic and unidentified, mCherry mRNA-expressing neurons was quite even throughout the NI. These findings should be taken into consideration in any future experiments involving intra-NI injections, especially involving genetically modified animals.

Previous studies have shown that NI-originating RLN3/GABA projections target GABA and glutamate neurons in the MS, whereas cholinergic MS neurons lack RXFP3 mRNA (Albert-Gascó et al., 2018; Haidar et al., 2019). In view of the current results, possible targeting of MS cholinergic neurons by glutamatergic axons originating from the NI should be examined, particularly as it is known that activation of MS cholinergic cells promotes locomotor activity and increases hippocampal theta power (Lu et al., 2020), and similar effects are observed after activation of the NI (Ma et al., 2017a). Thus, the relative role of GABA vs. glutamate released from NI neurons emerges as an important issue in the control of septohippocampal network activity and related processes, and warrants further investigation in different species.

Most excitatory neurons in the central nervous system appear to express a vesicular transporter for the fast neurotransmitter, glutamate (vGlut1–3), but some glutamatergic neurons also express the vesicular GABA transporter vGAT, an example being a distinct group of neurons in the lateral supramammillary nucleus (SuML) (Soussi et al., 2010), an area critically involved in hippocampal theta rhythm control via direct connections with the septum and hippocampus (Borhegyi et al., 1997; Kiss et al., 2000). The net action of GABA/glutamate co-transmission from SuML to hippocampus was shown to be excitatory (Hashimotodani et al., 2018) and activation of the SuML-hippocampal axis activates dentate gyrus (DG) neurons and increases DG theta and gamma power during paradoxical sleep (Billwiller et al., 2020). The rat NI is directly and reciprocally connected with the medial and lateral parts of the SuM (Goto et al., 2001; Olucha-Bordonau et al., 2003), and similar to the SuM, theta phase-locked neurons were identified in the NI (Kirk and McNaughton, 1991; Ma et al., 2013; Li et al., 2020; Trenk et al., 2022). Our current studies revealed that in the NI a significant proportion of neurons display, like in SuML, a dual neurochemical phenotype for GABAergic and glutamatergic neurotransmission, as both vGlut2 and vGAT1 mRNA is present in >20% of NI neurons innervating the rat MS, but the net action of this input remains unclear. Given the theta promoting effect of NI activation (Nuñez et al., 2006; Ma et al., 2017a; Lu et al., 2020), it is tempting to speculate that, similar to the dual GABA/glutamate transmission from SuML to hippocampus, the net effect of this mixed input from the NI to MS input is excitatory, and this excitatory effect could underlie the observed increase in hippocampal theta rhythm power after NI activation. The GABAergic transmission in this dual-phenotype pathway, may be critical for setting MS neuronal excitability and adjusting it to the current behavioral state, as it was shown for lateral habenula neurons in mice (Shabel et al., 2014; Meye et al., 2016). Therefore, coexistence of vGlut2 and vGAT and resultant excitatory and inhibitory transmission in the same neurons may underlie the fine tuning of the rhythmic neuronal activity within their target structures, and may be an important element of the neuronal mechanisms involved in synchronization of hippocampal theta rhythm and associated cognitive functions.

Earlier studies revealed that different MS neurons innervate dorsal and ventral hippocampus (Yoshida and Oka, 1995), areas implicated in memory/spatial navigation and emotional processing, respectively (reviewed in Fanselow and Dong, 2010). Given the involvement of the NI in emotional behaviors and stress (Banerjee et al., 2010; Ma et al., 2013; Blasiak et al., 2017; Olucha-Bordonau et al., 2018), and current results demonstrating that a substantial proportion of NI neurons innervating the MS express CRHR1 receptors, the involvement of different NI populations that are differentially sensitive to stress hormones, in specified MS neuronal circuits is a distinct possibility.

Our immunohistochemical studies revealed that RLN3 and CCK neurons are largely separate populations and account for approximately two-thirds of NI GABAergic neurons, representing ∼50 and 20%, respectively. CCK can therefore be considered a marker for a proportion of the GABAergic, non-RLN3 neuron population in the NI. Since in our *in situ* hybridization experiments, we did not assess CCK mRNA expression, neurons in which we detected only vGAT1 mRNA may belong to the CCK neuron population. At the same time these neurons may express NMB, as Lu et al. (2020) reported that in mice half of the NMB-positive NI neurons constitute a distinct population from the RLN3-positive neurons. However, it is not known whether NMB and CCK are, at least in part, colocalized in individual NI neurons, and further research is needed to address this question in both rats and mice.

The functional importance of the CCK neurons located within the NI has not been explored experimentally, although a study that investigated the role of CCK projections to the paraventricular nucleus of the rat thalamus in stress responses identified a population of CCK neurons in a region nominated as the “caudal dorsal raphé nucleus” (Bhatnagar et al., 2000), which were, in fact, CCK neurons of the NI. In support of this idea, the authors noted that the CCK neurons were only observed in the caudal aspects of the “dorsal raphé,” and were absent from anterior-mid levels of this nucleus (Bhatnagar et al., 2000). In contrast, although we observed that CCK-positive neurons tend to cluster medially in the NIc, they are distributed throughout the rostrocaudal extent of the NI. Notably, these CCK neurons were reported to exert a modulatory influence on behavioral components of stress-adaptation responses via the thalamic paraventricular nucleus, and to influence the function of the hypothalamic–pituitary–adrenal (HPA) axis in response to chronic stress (Bhatnagar et al., 2000).

The profile of calcium-binding protein expression in rat NI neurons suggests, contrary to speculation that calbindin and calretinin represent markers of separate neuronal populations in the NI (Cervera-Ferri et al., 2012), that the two proteins display a moderate degree of co-localization and are both present in RLN3- and CCK- positive and negative neurons. The broad pattern of co-localization of RLN3, with and without calbindin and calretinin, reinforces the complex heterogeneity of NI RLN3 neurons. In contrast, the majority of CCK neurons co-express both calbindin and calretinin, although a substantial population expresses neither protein. Further studies are required to assess the functional significance of calcium-binding protein utilization in specific NI populations, based on projection targets, afferent inputs, and/or intrinsic firing properties.

In our morphological studies, an interesting feature observed in about one third of NI neurons studied was the presence of varicose dendrites directed toward the fourth ventricle. These dendrites may release neuropeptides into the cerebrospinal fluid (CSF), as observed for hypothalamic neurons synthetizing vasopressin, oxytocin, GnRH, and other neuropeptides (Caraty et al., 2002; Ludwig and Leng, 2006). Notably, the majority of RLN3 neurons examined had this dendritic morphology. Therefore, RLN3 may be released into the CSF and influence structures that are not strongly innervated by the NI, or other RLN3 sources, but are enriched in cognate receptors for RLN3 (i.e., RXFP3). One such structure is the paraventricular nucleus of the hypothalamus (PVN), which is virtually devoid of RLN3 fibers, but has the highest density of RXFP3 expression in the rat brain (Kania et al., 2020b).

The dendrites of NI neurons, which form close appositions with the layer of tanycytes lining the fourth ventricle, may also be influenced by substances circulating in the CSF. We and others have shown that the tanycytes in the wall of the ventricle adjacent to the NI contain melanin-concentrating hormone (MCH) (Torterolo et al., 2008; Sabetghadam et al., 2018), and transport of this peptide may be mediated by both classical axonal transport and by the CSF (Noble et al., 2018).

Our current electrophysiological recordings revealed that the MS is almost exclusively innervated by previously described type I NI neurons, expressing a low-activation threshold potassium A-current (I_A_) (Blasiak et al., 2015). A comparison of the electrophysiological and neurochemical properties of type I and II NI neurons, the latter characterized by a calcium-dependent rebound depolarization at the offset of the hyperpolarizing current pulse (Blasiak et al., 2015), identified significant differences in the electrophysiology and neurochemistry of these cells.

Among the electrophysiological features distinguishing type I from type II neurons was the regular, repetitive firing at low frequencies, characteristic for all type I neurons, as well as their low excitability. Both of these characteristics of type I neurons may be a consequence of the activation of I_A_ currents (Hille, 2001; Zemel et al., 2018). Functionally, activation of the I_A_ current in NI neurons innervating the MS may be an element of the fine tuning of the inputs that reach the NI and the resulting selection and integration of information subsequently transmitted to the MS, through the process known as subtractive inhibition (Goldwyn et al., 2018). At the same time, A-type potassium current-dependent divisive inhibition and consequential gain control (Silver, 2010), may underlie the lower firing rate of type I than type II NI neurons in response to depolarizing inputs, as we have not detected the presence of an I_A_ current in the latter population.

Apart from their lower excitability than type II cells, type I NI neurons were, in general, more inhibited and displayed a lower sensitivity to the excitatory drive state, which was manifested by their lower membrane potential, lower spontaneous firing frequency, longer time to first spike in response to depolarization and a much higher value of the rheobase than in type II neurons. These characteristics may result from both intrinsic passive and active properties of type I neuron membranes and from synaptic inputs, which in the case of type I neurons were primarily inhibitory, with a higher frequency and amplitude than in type II neurons. Heterogeneity of NI neurons was also manifested by different sag properties, indicative of different levels of hyperpolarization activated cationic (HCN) channel expression (Zolles et al., 2006). We observed that type I neurons have a higher sag amplitude, which may underlie the regularity of their firing pattern (Chan et al., 2004).

The shape of action potentials is a unique feature of neuronal populations, and reflects their information processing abilities (Bean, 2007), and the differing shape of the action potentials of type I and II NI neurons may reflect their expression of specific ion channels. Neuronal AHP is a multicomponent phenomenon that can be divided into fast, medium, and slow AHP (fAHP, mAHP, and sAHP, respectively), on the basis of temporal and voltage dynamics, as well as on the basis of the underlying currents. fAHP lasts 1–10 ms, occurs immediately after an action potential, and depends on the activation of the large conductance calcium-activated potassium channels (BK) channels. mAHP also has a fast onset (less than 10 ms), but can last for hundreds of milliseconds (typically 50–200 ms) and can depend on a large variety of channels, including small conductance calcium-activated potassium (SK), A-type potassium, Kv7/KCNQ/M, and HCN/h-channels. sAHP peaks between 400 and 700 ms after a single or a train of action potentials, and lasts from 0.5 s to several seconds. In some neurons, afterdepolarization (ADP) mediated by inward currents facilitating burst generation may occur between fAHP and mAHP (Yue and Yaari, 2004; Gu et al., 2005; Kim et al., 2005; Bean, 2007). Based on these criteria, we classified the AHP of type I NI neurons as type I mAHP, and AHPs observed in type II neurons as fAHP and type II mAHP. Importantly, type I mAHP differed in terms of trough timing and amplitude, from all types of AHPs observed in type II NI neurons. It is possible that rapidly activating I_A_ currents are expressed by type I NI neurons and participate in shaping the AHP in this neural population, as it was shown that activation of the I_A_ current during an action potential, increases AHP trough amplitude (D’Angelo et al., 1998; Kim et al., 2005; Stern et al., 2015). Although the specific ionic mechanisms underlying type I and type II neuron AHPs need to be verified, the observation in the present study of distinctive low trough values of mAHP in type I cells, along with the absence of ADP, allows for an unequivocal differentiation of these neurons *ex vivo*. Moreover, AP shape differences along with differences in passive membrane properties, namely membrane capacity and time constant, further indicate intrinsic differences between the examined NI neuron populations.

Despite differences in active and passive membrane properties, the types of NI neurons investigated did not differ in morphology, indicating that the observed differences resulted, at least in part, from expression of specific ion channels in the cell membrane of type I and II neurons. Dichotomous cellular properties of NI neurons were also manifested in the selective expression of RLN3 and CCK neuropeptides in type I cells. Our results also demonstrate that type I NI neurons, distinct from type II neurons, can form neuronal networks that project to specific brain areas, suggesting that they serve distinct functional roles. This is supported by results of our recent *in vivo* study using projection specific opto-tagging, revealing that only regular, fast-firing theta phase-independent NI neurons (type I *ex vivo*) innervate the MS, whereas a distinct population of theta bursting NI neurons, receives direct innervation from the MS (Trenk et al., 2022). Further studies are needed to unequivocally determine whether MS innervated NI cells that burst under *in vivo* conditions, belong to the type II neurons described in the current study, but cited *in vivo* and *ex vivo* characteristics [bursting phenotype and the expression of burst-firing patterns underlying, putative T-type calcium currents (Arshaad et al., 2021), respectively], support this conclusion. It can therefore be assumed that distinctive intrinsic properties and synaptic inputs to type I NI neurons innervating the MS, interact to select and propagate information that influences MS-dependent theta rhythm and related behaviors (Nuñez et al., 2006; Ma et al., 2009, 2013; Lu et al., 2020; Gil-Miravet et al., 2021). These findings will assist in efforts to further dissect specific NI-associated neural circuits, and highlight the opportunity to obtain new knowledge from investigations of this small enigmatic nucleus and its role in both the functioning of healthy forebrain circuits and in their neuropathology.

## Data Availability Statement

The raw data supporting the conclusions of this article will be made available by the authors, without undue reservation.

## Ethics Statement

The animal studies were reviewed and approved by the 2nd Local Institutional Animal Care and Use Committee (Krakow, Poland), approval number 24/2021, and The Florey Institute of Neuroscience and Mental Health Animal Ethics Committee.

## Author Contributions

AB, TB, SM, and ALG conceived the project and contributed to the experimental design. AB supervised all aspects of the work, and performed, analyzed, and interpreted the *ex vivo* electrophysiology data, and drafted the manuscript. PS performed viral injections and patch-clamp experiments with optical stimulation. AT performed the neural tract-tracing experiments. AG and AT performed the RNAscope *in situ* hybridization assays. AG performed the Sholl analysis. AT, CS, and SM performed immunohistochemical staining, and analyzed and interpreted resultant microscopy data. AS, PS, AT, AG, CS, and GD created the figures. AB and ALG revised the manuscript and figures. All authors provided comments and corrections, provided approval for publication of the content and agreed to be accountable for all aspects of the work.

## Conflict of Interest

The authors declare that the research was conducted in the absence of any commercial or financial relationships that could be construed as a potential conflict of interest.

## Publisher’s Note

All claims expressed in this article are solely those of the authors and do not necessarily represent those of their affiliated organizations, or those of the publisher, the editors and the reviewers. Any product that may be evaluated in this article, or claim that may be made by its manufacturer, is not guaranteed or endorsed by the publisher.
