# Supplementary material for: Functional Neuroanatomy of the Rat Nucleus Incertus–Medial Septum Tract: Implications for the Cell-Specific Control of the Septohippocampal Pathway
Source: Front Cell Neurosci. 2022 Feb 25;16:836116. doi: 10.3389/fncel.2022.836116 (PMC8913896; doi:10.3389/fncel.2022.836116)
Supplement: Supplementary file 1 [file Table_1.DOCX]

Supplementary Material

**Supplementary Table 1. Primary antibodies**.

| **Antigen** | **Description of Immunogen** | **Source/Cat No.** | **Dilution** | **RRID** |
| --- | --- | --- | --- | --- |
| Relaxin-3 (RLN3) | N-terminal region of mature RLN3 peptide conserved in rat and human | Florey Institute of Neuroscience and Mental Health, Parkville, Australia, mouse monoclonal | 1:5 or 1:25 | AB_2315266 |
| Calbindin (CB) | C-terminus of calbindin D28K of human origin | Santa Cruz (Santa Cruz, CA, USA), sc-7691, goat polyclonal | 1:1000 | AB_634520 |
| Calretinin (CR) | Recombinant human calretinin containing a 6-his tag at the N-terminal | Swant (Marly, Switzerland), 7697, rabbit polyclonal | 1:1000 | AB_2721226 |
| Calretinin (CR) | Epitope within first 4 EF-hands domains common to human calretinin and calretinin-22K | Swant, 6B3, mouse monoclonal | 1:2500 | AB_10000320 |
| Cholecystokinin (CCK) | Synthetic sulfated CCK-8 conjugated to KLH | Sigma-Aldrich (St. Louis, MO, USA), C2581, rabbit polyclonal | 1:2000 or 1:4000 | AB_258806 |
| Pro–cholecystokinin (pro-CCK) | Mouse CCK aa 107-115 (C-terminal 9 amino acids) | Frontier Institute Co., Ltd (Hokkaido, Japan), CCK-pro-RB-Af350, rabbit polyclonal | 1:200 | AB_2571674 |
| γ-Aminobutyric acid (GABA) | γ-Aminobutyric acid conjugated to BSA | Sigma-Aldrich, A2052, rabbit polyclonal | 1:6000 | AB_477652 |
| Glutamic acid decarboxylase 65/67 (GAD-6) | Full length protein of purified glutamic acid decarboxylase from rat brain | Developmental Studies Hybridoma Bank (University of Iowa, IA, USA) GAD-6, mouse monoclonal | 1:200 | AB_2314499 |
| Neuronal nuclei (NeuN) | Purified cell nuclei from mouse brain | Merck Millipore (Bayswater, VIC, Australia), MAB377, mouse monoclonal | 1:1000 | AB_2298772 |

**Supplementary Table 2. Secondary antibodies**.

| **Antibody** | **Conjugate** | **Source/Cat. No.** | **RRID** |
| --- | --- | --- | --- |
| Donkey anti-mouse | Alexa Fluor 488 | Jackson ImmunoResearch (West Grove, PA, USA) / 715-545-151 | AB_2341099 |
| Donkey anti-rabbit | Alexa Fluor 594 | Jackson ImmunoResearch / 711-585-152 | AB_2340621 |
| Donkey anti-mouse | Alexa Fluor 594 | Jackson ImmunoResearch / 715-585-151 | AB_2340855 |
| Donkey anti-mouse | Alexa Fluor 647 | Jackson ImmunoResearch / 715-605-151 and 715-606-150 | AB_2340863 and AB_2340865 |
| Donkey anti-rabbit | Alexa Fluor 647 | Life Technologies (Carlsbad, CA, USA) / A-31573 | AB-2536183 |
| Goat anti-rabbit | Alexa Fluor 594 | Life Technologies / A-11012 | AB_2534079 |
| Goat anti-mouse | DyLight 405 | Jackson ImmunoResearch / 115-475-003 | AB_2338786 |
| Donkey anti-sheep | Alexa Fluor 488 | Jackson ImmunoResearch / 713-545-147 | AB_2340745 |
| Donkey anti-goat | Alexa Fluor 594 | Life Technologies / A-11058 | AB_2534105 |
| Donkey anti-rabbit | Alexa Fluor 488 | Jackson ImmunoResearch / 711-546-152 and 711-545-152 | AB_2340619 and AB_2313584 |

**Supplementary Table 3. Quantification of RLN3, calbindin and calretinin colocalization in rat NI**.

| **Total neurons** | **Mean neuron count** | **SEM** |
| --- | --- | --- |
| RLN3 | 78.96 | 5.61 |
| CR | 126.75 | 11.76 |
| CB | 93.68 | 7.81 |

| **Neuron type** | **Mean neuron count** | **SEM** | **Approximate proportion of total counted neurons (%)** |
| --- | --- | --- | --- |
| RLN3, CR and CB | 32.46 | 3.66 | 18 |
| RLN3 and CR | 19.61 | 1.80 | 11 |
| RLN3 and CB | 11.96 | 1.21 | 7 |
| CR and CB | 26.89 | 4.44 | 14 |
| RLN3 only | 14.93 | 1.61 | 9 |
| CR only | 47.79 | 3.77 | 28 |
| CB only | 22.36 | 2.29 | 13 |

| **Pairwise comparisons** | **Colocalization** | **SEM** |
| --- | --- | --- |
| RLN3/CR | 0.41 | 0.02 |
| CR/RLN3 | 0.65 | 0.02 |
| RLN3/CB | 0.48 | 0.02 |
| CB/RLN3 | 0.56 | 0.02 |
| CR/CB | 0.61 | 0.03 |
| CB/CR | 0.45 | 0.03 |

**Supplementary Table 4. Quantification of CCK, calbindin and calretinin colocalization in rat NI**.

| **Total neurons** | **Mean neuron count** | **SEM** |
| --- | --- | --- |
| CCK | 33.28 | 2.70 |
| CR | 102.22 | 6.11 |
| CB | 105.69 | 7.45 |

| **Neuron type** | **Mean neuron count** | **SEM** | **Approximate proportion of total counted cells (%)** |
| --- | --- | --- | --- |
| CCK, CR and CB | 15.50 | 1.61 | 9 |
| CCK and CR | 3.31 | 0.45 | 2 |
| CCK and CB | 5.42 | 0.64 | 3 |
| CR and CB | 31.81 | 3.42 | 18 |
| CCK only | 9.06 | 1.65 | 6 |
| CR only | 51.61 | 2.88 | 31 |
| CB only | 52.97 | 3.51 | 31 |

| **Pairwise comparisons** | **Colocalization** | **SEM** |
| --- | --- | --- |
| CCK/CR | 0.18 | 0.01 |
| CR/CCK | 0.58 | 0.04 |
| CCK/CB | 0.20 | 0.01 |
| CB/CCK | 0.64 | 0.04 |
| CR/CB | 0.43 | 0.02 |
| CB/CR | 0.45 | 0.03 |

**Supplementary Table 5. Sholl analysis of type I and II NI neurons**.

| **Distance from soma radius [µm]** | **Type I** | | | **Type II** | | | **Two-way RM ANOVA** | | | | **Post hoc Uncorrected Fisher's LSD test (p value)** |
| --- | --- | --- | --- | --- | --- | --- | --- | --- | --- | --- | --- |
|  |  |  |  |  |  |  | **Distance from soma** | **Type** | **Interaction** | **Subjects (matching)** |  |
| 10 | 2.29 | ± | 1.37 | 2.71 | ± | 1.68 | **p < 0.0001** | 0.94 | **p = 0.003** | **p < 0.0001** | 0.53 |
| 20 | 4.96 | ± | 1.97 | 3.86 | ± | 2.74 |  |  |  |  | 0.11 |
| 30 | 5.83 | ± | 2.71 | 3.93 | ± | 2.46 |  |  |  |  | **0.01** |
| 40 | 5.63 | ± | 3.00 | 4.71 | ± | 2.46 |  |  |  |  | 0.18 |
| 50 | 5.92 | ± | 4.17 | 4.86 | ± | 2.35 |  |  |  |  | 0.12 |
| 60 | 5.50 | ± | 3.36 | 4.79 | ± | 2.33 |  |  |  |  | 0.29 |
| 70 | 5.25 | ± | 3.11 | 4.43 | ± | 1.99 |  |  |  |  | 0.23 |
| 80 | 5.00 | ± | 3.06 | 4.36 | ± | 1.91 |  |  |  |  | 0.34 |
| 90 | 4.71 | ± | 3.14 | 4.07 | ± | 1.73 |  |  |  |  | 0.35 |
| 100 | 4.33 | ± | 2.88 | 3.86 | ± | 1.88 |  |  |  |  | 0.48 |
| 110 | 3.71 | ± | 2.54 | 3.79 | ± | 1.76 |  |  |  |  | 0.91 |
| 120 | 3.33 | ± | 2.35 | 3.36 | ± | 1.74 |  |  |  |  | 0.97 |
| 130 | 3.13 | ± | 2.56 | 3.00 | ± | 1.66 |  |  |  |  | 0.85 |
| 140 | 3.08 | ± | 2.45 | 3.43 | ± | 2.31 |  |  |  |  | 0.61 |
| 150 | 2.75 | ± | 2.45 | 2.86 | ± | 1.66 |  |  |  |  | 0.87 |
| 160 | 2.79 | ± | 2.43 | 2.71 | ± | 1.64 |  |  |  |  | 0.91 |
| 170 | 2.50 | ± | 2.30 | 3.00 | ± | 2.04 |  |  |  |  | 0.46 |
| 180 | 2.17 | ± | 1.95 | 2.64 | ± | 1.78 |  |  |  |  | 0.48 |
| 190 | 2.08 | ± | 1.77 | 2.14 | ± | 1.61 |  |  |  |  | 0.93 |
| 200 | 1.83 | ± | 1.79 | 2.07 | ± | 1.90 |  |  |  |  | 0.73 |
| 210 | 1.63 | ± | 1.66 | 2.14 | ± | 1.99 |  |  |  |  | 0.45 |
| 220 | 1.46 | ± | 1.53 | 2.64 | ± | 3.84 |  |  |  |  | 0.08 |
| 230 | 1.54 | ± | 1.72 | 1.86 | ± | 1.66 |  |  |  |  | 0.64 |
| 240 | 1.33 | ± | 1.46 | 1.86 | ± | 1.79 |  |  |  |  | 0.44 |
| 250 | 1.29 | ± | 1.49 | 1.64 | ± | 1.82 |  |  |  |  | 0.61 |
| 260 | 1.13 | ± | 1.39 | 1.71 | ± | 1.94 |  |  |  |  | 0.39 |
| 270 | 0.96 | ± | 1.20 | 1.79 | ± | 2.08 |  |  |  |  | 0.22 |
| 280 | 0.96 | ± | 1.20 | 1.71 | ± | 2.16 |  |  |  |  | 0.27 |
| 290 | 0.96 | ± | 1.20 | 1.71 | ± | 2.16 |  |  |  |  | 0.27 |
| 300 | 0.83 | ± | 1.20 | 1.50 | ± | 1.79 |  |  |  |  | 0.33 |
| 310 | 0.83 | ± | 1.17 | 1.43 | ± | 1.83 |  |  |  |  | 0.38 |
| 320 | 0.75 | ± | 1.07 | 1.21 | ± | 1.53 |  |  |  |  | 0.49 |
| 330 | 0.67 | ± | 0.92 | 2.00 | ± | 3.94 |  |  |  |  | **0.0497** |
| 340 | 0.63 | ± | 0.82 | 0.71 | ± | 0.91 |  |  |  |  | 0.90 |
| 350 | 0.63 | ± | 0.82 | 0.57 | ± | 0.94 |  |  |  |  | 0.94 |
| 360 | 0.63 | ± | 0.82 | 0.43 | ± | 0.65 |  |  |  |  | 0.77 |
| 370 | 0.63 | ± | 0.82 | 0.43 | ± | 0.76 |  |  |  |  | 0.77 |
| 380 | 0.58 | ± | 0.78 | 0.29 | ± | 0.61 |  |  |  |  | 0.66 |
| 390 | 0.54 | ± | 0.72 | 0.21 | ± | 0.58 |  |  |  |  | 0.63 |
| 400 | 0.50 | ± | 0.78 | 0.21 | ± | 0.58 |  |  |  |  | 0.67 |

Data are presented as mean ± SD.

**Supplementary Table 6. Comparison of the morphological parameters of type I and type II NI neurons**.

| **Morphological parameter** | **Type I** | **Type II** | **Mann-Whitney test** |
| --- | --- | --- | --- |
| Total dendritic length [µm] | 997 ± 837 | 1179 ± 1077 | p = 0.65 |
| Number of primary dendrites | 3.00 ± 1.75 | 3.00 ± 2.00 | p = 0.38 |
| Number of branches | 14.00 ± 11.75 | 17.00 ± 14.50 | p = 0.88 |
| Number of bifurcations | 6.00 ± 5.75 | 7.00 ± 7.25 | p = 0.64 |
| Number of dendritic tips | 8.50 ± 6.00 | 10.00 ± 7.25 | p = 0.99 |

Data are presented as median ± interquartile range.
